# Development and validation of prognostic risk prediction models for hepatocellular carcinoma patients treated with immune checkpoint inhibitors based on a systematic review and meta-analysis of 47 cohorts

**DOI:** 10.3389/fimmu.2023.1215745

**Published:** 2023-07-14

**Authors:** Delin Ma, Mingkun Liu, Xiangyu Zhai, Xianzhi Li, Bin Jin, Yang Liu

**Affiliations:** ^1^ Department of Hepatobiliary Surgery, Peking University People’s Hospital, Beijing, China; ^2^ Department of Organ Transplantation, Qilu Hospital, Cheeloo College of Medicine, Shandong University, Jinan, China; ^3^ Department of General Surgery, The Second Hospital of Shandong University, Jinan, China; ^4^ Hepatobiliary Surgery Research Center of Shandong University, Jinan, China; ^5^ Renal Division, Peking University First Hospital, Beijing, China; ^6^ Department of General Surgery, Vascular Surgery, Shandong University Qilu Hospital, Jinan, China

**Keywords:** hepatocellular carcinoma, immune checkpoint inhibitors, meta-analysis, prediction model, prognosis

## Abstract

**Objective:**

To identify the risk factors associated with prognosis in patients with hepatocellular carcinoma (HCC) treated with immune checkpoint inhibitors (ICI) via meta-analysis. And to construct prediction models to aid in the prediction and improvement of prognosis.

**Methods:**

We searched PubMed, Embase, Web of Science and Cochrane Library for relevant studies from inception to March 29, 2023. After completing literature screening and data extraction, we performed meta-analysis, sensitivity analysis, and subgroup analysis to identify risk factors associated with OS and PFS. Using the pooled hazard ratio value for each risk factor, we constructed prediction models, which were then validated using datasets from 19 centers in Japan and two centers in China, comprising a total of 204 patients.

**Results:**

A total of 47 studies, involving a total of 7649 ICI-treated HCC patients, were included in the meta-analysis. After analyzing 18 risk factors, we identified AFP, ALBI, NLR, ECOG performance status, Child-Pugh stage, BCLC stage, tumor number, vascular invasion and combination therapy as predictors for OS prediction model, while AFP, ALBI, NLR, ECOG performance status, Child-Pugh stage, BCLC stage, tumor number and vascular invasion were selected as predictors for PFS model. To validate the models, we scored two independent cohorts of patients using both prediction models. Our models demonstrated good performance in these cohorts. In addition, in the pooled cohort of 204 patients, Our models also showed good performance with area under the curve (AUC) values of 0.712, 0.753, and 0.822 for the OS prediction model at 1-year, 2-year, and 3-year follow-up points, respectively, and AUC values of 0.575, 0.749 and 0.691 for the PFS prediction model Additionally, the calibration curve, decision curve analysis, and Kaplan-Meier curves in the pooled cohort all supported the validity of both models.

**Conclusion:**

Based on the meta-analysis, we successfully constructed the OS and PFS prediction models for ICI-treated HCC patients. We also validated the models externally and observed good discrimination and calibration. The model’s selected indicators are easily obtainable, making them suitable for further application in clinical practice.

## 1 Introduction

Primary liver cancer is the sixth most commonly diagnosed cancer and the third leading cause of cancer-related death globally, of which hepatocellular carcinoma (HCC) accounts for 75%-85% of all cases (1). HCC often lacks symptoms in its early stages, leading to diagnosis at an intermediate or advanced stage, where non-surgical treatment is preferred (2). Before the advent of immunotherapy, tyrosine kinase inhibitors (TKI) such as sorafenib were the first choice for the treatment of advanced HCC, but their efficacy in advanced HCC was not ideal (3–5). In recent years, substantial advancements have been achieved in the immunotherapy and second-line treatments of liver cancer (6). Immune checkpoint inhibitors (ICI) represent a classic form of immunotherapy that target typical immune checkpoints, such as programmed death 1 (PD-1), programmed death ligand 1 (PD-L1), and cytotoxic T-lymphocyte-associated antigen 4 (CTLA-4). These drugs counteract the immunosuppressive action of these checkpoints, restoring the T cells’ function to exert anti-tumor activity (7). Some clinical trials, including NCT03434379 and NCT03794440, have reported that the combination of ICI and TKI significantly prolonged overall survival (OS) and progression-free survival (PFS) in patients with HCC (5, 8–10), and some studies showed that some ICIs could be used as novel second-line agents in the treatment of HCC (6, 11). As a result, Atezolizumab and bevacizumab therapy in combination has become the primary systemic treatment for unresectable HCC in Asian countries (12). However, their therapeutic efficacy can be affected by many factors, such as the level of tumor immunogenicity, characteristics of the tumor microenvironment, physical conditions of patients (13). Therefore, not all patients can benefit from ICI treatment. Therefore, how to screen patients who may benefit from ICI has become an urgent problem to be solved.

Previous studies had found that some clinical indicators may be correlated with clinical outcomes of HCC patients receiving ICI treatment. For instance, some studies found AFP response may be associated with better OS and PFS for unresectable HCC, and other studies showed that ECOG Performance Status and Child-Pugh stage also had predictive efficacy (14–16). In recent years, tumor-related inflammatory responses, including local and systemic inflammation, are regarded as significant contributors to the development and progression of malignant tumors (17, 18). Some inflammatory markers such as neutrophil-to-lymphocyte ratio (NLR), platelet-to-lymphocyte ratio (PLR) and C-reactive protein levels are valuable indicators that reflect both the magnitude of inflammatory response and immune status. Recent investigations have demonstrated the efficacy of these inflammatory markers in predicting tumor prognosis and their association with poor OS or PFS in multiple types of cancer. Among the markers, NLR has been found to be a valuable indicator for predicting the outcome of ICI treatment across various types of cancer (19–21). Many previous studies suffer from limitations such as small sample sizes, a lack of external validation, and being confined to single-center series. To address these drawbacks, this study conducts a meta-analysis of relevant cohort studies to identify the risk factors influencing the prognosis of HCC patients undergoing ICI treatment. Furthermore, clinical prediction models will be constructed to provide guidance for clinical decision-making.

## 2 Methods

### 2.1 Meta-analysis section

#### 2.1.1 Literature search strategy

This study followed the Preferred Reporting Items for Systematic Reviews and Meta-analyses (PRISMA) to conduct the meta-analysis. Two authors (MDL, LMK) independently searched for relevant studies from four databases: PubMed, Embase, Web of Science, and Cochrane Library. The literature search had no predefined start date and was updated until March 29, 2023. Further details on the search strategy are provided in the **Supplemental File Section 1**.

#### 2.1.2 Screening criteria

Studies that met the following criteria were included in this study (1): Patients diagnosed with HCC, excluding intrahepati cholangiocacinoma and combined hepatocellular carcinoma and cholangiocarcinoma (cHCC-CC) (2); receipt of ICI treatment, including PD-1, PD-L1, or CTLA-4 inhibitors (3); investigation of at least one risk factor associated with prognosis (4); reported OS and PFS (5); provided sufficient information to assess hazard ratios (HR) with 95% confidence intervals (95% CI) (6); studies were published in English. Studies that met the following criteria were excluded (1). review studies, meta-analyses, and case reports (2); basic experimental studies of HCC or studies not related to the subject of this study (3); insufficient data reported to extract relevant information for analysis. If multiple studies describing the same outcome in the same population were available, only the most complete or recent one was included.

#### 2.1.3 Data extraction and quality assessment

Required data from the eligible studies were extracted independently by two authors (MDL and LXZ) and in cases of disagreement, a third author (LXZ) arbitrated. The following baseline characteristic data were extracted from the included studies: first author, year of publication, country of the study, study type (prospective/retrospective), sample size, age (median/mean), ICI type, and duration of follow-up (median). The quality of the included studies was assessed using the Newcastle-Ottawa Scale (NOS) criteria.

### 2.2 Development and validation of PFS and OS prediction model for HCC patients treated with ICI

#### 2.2.1 Construction of the prediction model

According to the results of Meta-analysis, the risk factors with statistical significance were preliminarily screened. Subsequently, the robustness of the pooled results of each risk factor was analyzed by sensitivity analysis, and only risk factors with stable sensitivity analysis results were used for the construction of the model. For ordered categorical variables, due to variations in the selection of appropriate cut-off values among different original studies, we determined them using the following method: for those with two options for cut-off value selection criteria, we took the one with the highest number of original studies as the standard. If there were three or more different cut-off values, we ranked all the selected cut-off values from low to high, and selected the median as the cut-off value for the model. Taking into account the racial differences and the regional characteristics of the validation cohort, we performed subgroup analysis based on region to achieve personalization and precision of the model. The β-coefficient for each risk factor was calculated from the pooled HR and 95%CI, using the formula β=ln (HR). The β-coefficient was then adjusted by multiplication by ten and rounding to one decimal place, following a method previously reported by Jiang et al. (22). The PFS and OS risk score tables for HCC patients treated with ICI were made, and the total score was the sum of the scores of each risk factor. Finally, the patient’s prognosis was determined based on the total score.

#### 2.2.2 Validation of the prediction model

**Figure 1:**
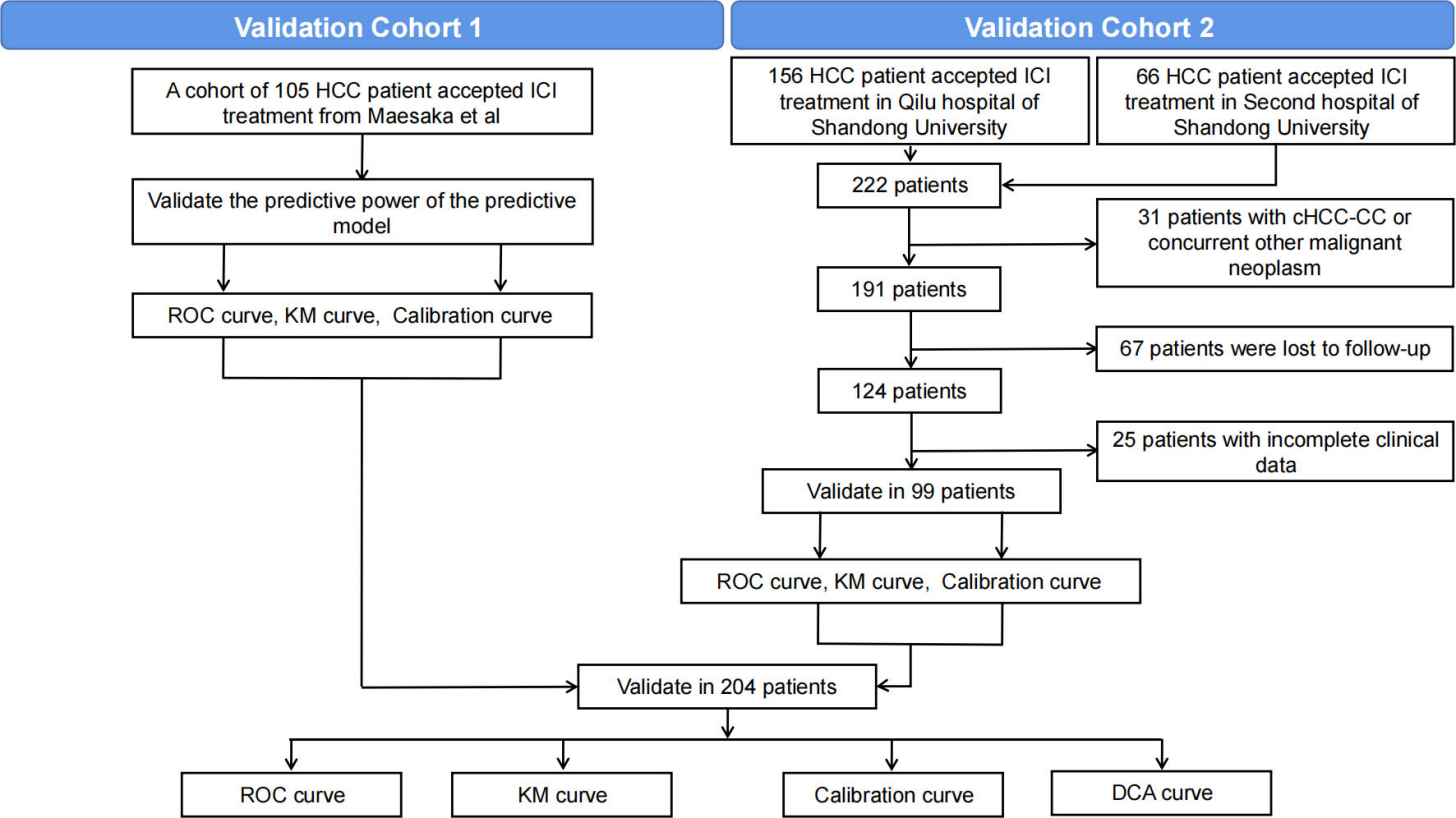
Process for the selection of patients in the 2 validation cohorts.

To evaluate the predictive performance of the model, we used two available cohorts for validation: validation cohort 1 (n=105) and validation cohort 2 (n=99). The overall validation process is shown in **Figure 1**. Validation cohort 1 was drawn from a multicenter study conducted by Maesaka et al., which retrospectively analyzed 105 HCC patients from 19 centers treated with atelelizumab plus bevacizumab as primary systemic therapy (23). The tumor number in this cohort used 5 as the cut-off point, and the detailed data on this variable were not available. Therefore, only this cut-off point could be utilized for subsequent model validation. Validation cohort 2 consisted of HCC patients receiving ICI treatment at Qilu Hospital of Shandong University and the Second Hospital of Shandong University from November 2018 to March 2023. Patient inclusion criteria (1): diagnosis of HCC based on clinical symptoms, serologic examination, imaging and pathologic assessment, and received at least one time ICI treatment (2); complete clinical data (3); follow-up information available. Exclusion criteria (1): cHCC-CC or concurrent other malignant neoplasm (2); missing clinical data (3); patients lost to follow-up. After screening, we excluded 32 patients with pathologically confirmed cHCC-CC or concurrent other malignancies, 49 patients lacking relevant clinical information, and 69 patients lacking follow-up information, resulting in the inclusion of 99 patients in the final validation cohort. The study adhered to the principles of the Declaration of Helsinki, and has been approved by both the Ethics Committees of Qilu Hospital of Shandong University and Second Hospital of Shandong University. Given that this study is a retrospective study, we have waived the requirement for informed consent and omitted any patient identification details to protect their privacy.

### 2.3 Statistical analysis

Heterogeneity was assessed using the Cochran’s Q test and Higgins inconsistency index (I^2). Significant heterogeneity was indicated by either a P value<0.1 or I^2>50% which required the use of a random-effects model; otherwise, a fixed-effects model was employed. Prior to further processing, the raw HR was log-transformed, and the metabin() function in R’s “meta” package was used for calculating meta-analytic effect size and its corresponding p-value. Publication bias was evaluated through Begg’s and Egger’s tests, and we applied the ‘trim and fill’ method for those pooled results that exhibited publication bias in both tests to assess the occurrence’s effect on pooled HR reliability. To test the robustness of our analyses, we performed sensitivity analyses for key findings through gradual deletion of the included studies one by one. When validating the model, we assessed its predictive performance using receiver operating characteristic (ROC) curves, calibration curves, and decision curve analysis (DCA) curves. In addition, we stratified patients into four groups (“low risk,” “medium risk,” “high risk,” and “very high risk”) based on their total scores’ first quartile, median, and third quartile, and then created Kaplan-Meier (KM) curves for each group to evaluate the model’s performance. The “DynNom” and “shiny” packages were used to construct web-based probability calculators, which could dynamically predict the probability of death and recurrence. All analyses were performed with R software (version 4.2.2). We considered P<0.05 as statistically significant unless otherwise specified.

## 3 Results

### 3.1 Meta-analysis section

#### 3.1.1 literature retrieval and screening results

**Figure 2:**
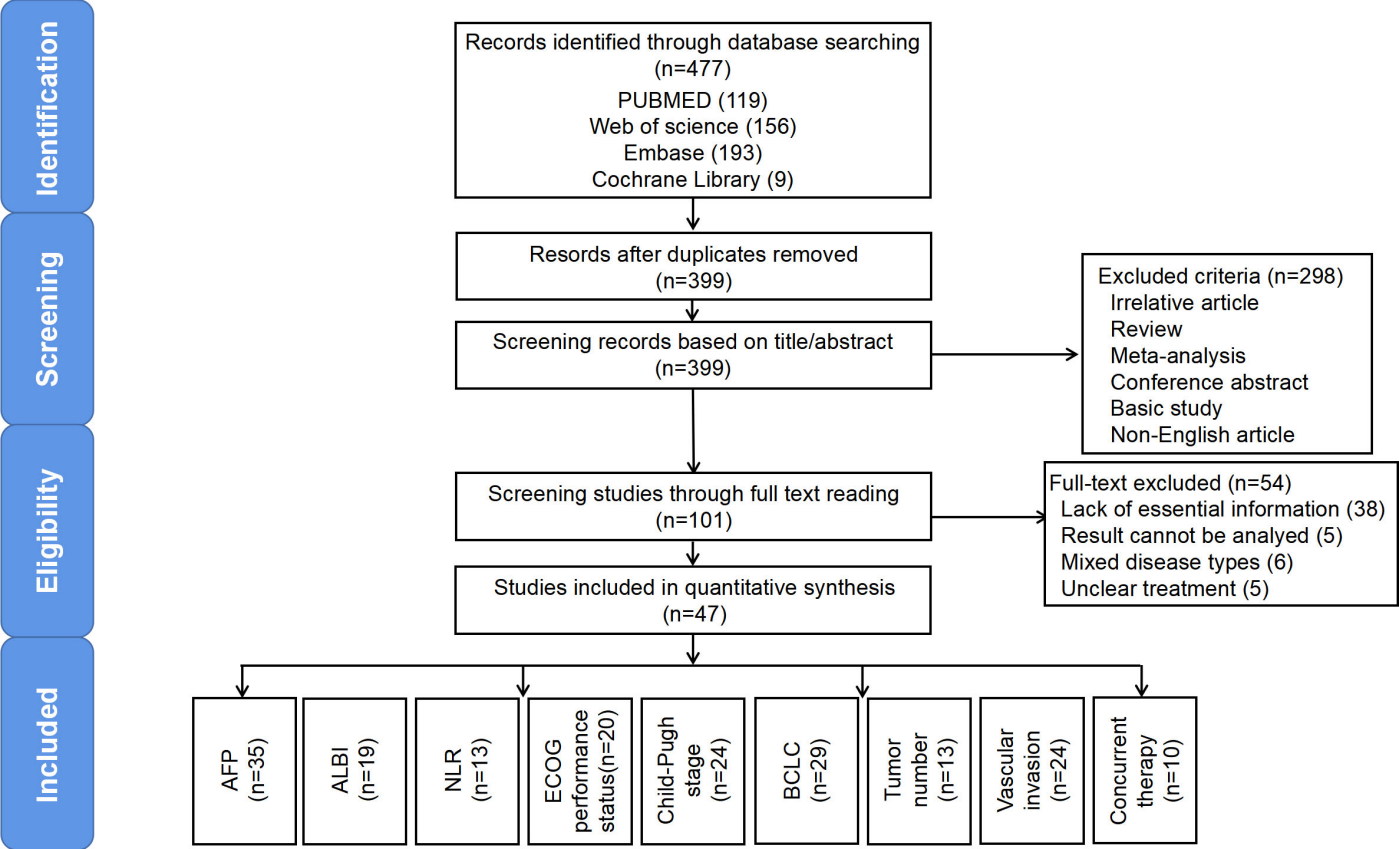
Flow diagram outlining the literature search and study selection for risk factors associated with the prognosis of HCC patients receiving ICI treatment.

A total of 479 studies were collected through searching the database, of which 129 were duplicates. Next, we excluded irrelevant studies, non-English studies, and those that were not clinical studies (i.e., basic studies, case reports, reviews, conference abstracts, systematic reviews, meta-analyses, etc.) by reading the titles and abstracts. After full-text screening of the remaining 101 studies, we ultimately included 47 in our meta-analysis. **Figure 2** further illustrates our process of study retrieval and screening.

A total of 47 studies were included. Published between 2019 and 2023, they were conducted in nine countries: 26 in China, 6 in Japan, 4 in the United States, 3 in South Korea, 2 each in the United Kingdom and Germany, and 1 each in Austria, Singapore, Italy, and France. There were 2 prospective studies and 45 retrospective studies. Forty-seven articles had a sample size ranging from 33 to 773, with a total sample size of 7649. Forty-three studies reported the mean or median age of the entire cohort, which ranged from 47 to 74 years. Forty-six studies reported the ICI class used, of which 29 used a PD-1 inhibitor (i.e., Nivolumab, Pembrolizumab, Camrelizumab), 13 used a PD-L1 inhibitor (Atezolizumab), and 4 had mixed ICI treatment. Thirty-one studies reported the median follow-up time, which ranged from 3.3 to 25.1 months. In terms of quality assessment of the included studies, the NOS scores of 47 studies ranged from 6 to 9, with 5 studies having a NOS score of 9, 16 studies having a NOS score of 8,22 studies having a NOS score of 7, and 4 studies having a NOS score of 6. Detailed baseline characteristics and quality scores for all included studies were provided in **Supplementary Tables 1**, **2**.

#### 3.1.2 Meta-analysis results

In this study, a total of 18 risk factors were included in the meta-analysis. And the result revealed that several factors were associated with OS, including sex, AFP, ALBI, BCLC stage, Child-Pugh stage, ECOG performance status, vascular invasion, tumor number, NLR and concurrent treatment. The pooled HR values were as follows:sex (0.89), AFP (1.51), ALBI (2.22), BCLC stage (1.40), Child-Pugh stage (2.03), ECOG performance status (2.26), vascular invasion (1.56), tumor number (1.63), NLR(1.41) and concurrent therapy (0.55). Additionally, AFP (HR 1.35), ALBI (HR 1.40), BCLC stage (HR 1.25), Child-Pugh stage (HR 1.38), ECOG performance status (HR 1.69), vascular invasion (HR 1.34), tumor number (HR 1.26), NLR (HR 1.34), PLR (HR 2.26) and concurrent therapy (HR 0.56) were correlated with PFS. In the sensitivity analysis, the pooled result of sex on OS and the pooled results of PLR and concurrent therapy on PFS were not stable. Subgroup analysis by region showed that there was a significant statistical difference (P<0.01) in the impact of ECOG scores on OS between Asian and non-Asian populations, while there were no differences observed in other aspects. Therefore, for the OS model, we will conduct a personalized prediction model of OS based on Asian/non-Asian. The results of the meta-analysis, sensitivity analysis and subgroup analysis are showed in **Supplementary Tables 3**, **5**, **Supplementary Figures 1**-**9**. In terms of publication bias, Begg’s test indicated a biased pooled analysis of BCLC stage (OS: P=0.01), vascular invasion (PFS: P=0.03), and NLR (OS: P=0.01). Similarly, Egger’s test revealed a biased pooled analysis of age (OS: P=0.01), BCLC stage (OS: P=0.01), ECOG performance status (OS: P=0.04), vascular invasion (PFS: P=0.04), tumor number (OS: P=0.03) and NLR (OS: P<0.01). However, the pooled results of BCLC stage, vascular invasion and NLR were not changed after correcting for publication bias, suggesting that the publication bias did not essentially affect the reliability of these pooled results. The detailed publication bias analysis results are showed in **Supplementary Tables 4**.

### 3.2 Development of the prediction models for OS and PFS

According to the results of meta-analysis, sensitivity analysis and subgroup analysis, we included 9 variables including AFP > 400 ng/ml (HR 1.51, 95%CI 1.37-1.66), ALBI >I (HR 2.22, 95%CI 1.95-2.53), NLR >3 (HR 1.41 95%CI 1.19-1.68), ECOG performance status >0 (Asian: HR 2.68, 95%CI 2.02-3.56, Non-Asian: HR 1.54, 95%CI 1.24-1.91), Child-Pugh stage >A (HR 2.03, 95%CI 1.62-2.53), BCLC stage >B (HR 1.4, 95%CI 1.23-1.58), tumor number >1 (HR 1.63, 95%CI 1.14-2.34), vascular invasion (HR 1.56, 95%CI 1.35-1.80), and concurrent therapy (HR 0.55, 95% CI 0.45-0.67) into the model for predicting OS, and their β coefficients were calculated as 0.41, 0.80, 0.34, 0.99 (Asian)/0.43 (Non-Asian), 0.71, 0.34, 0.49, 0.45 and -0.60. Meanwhile, the risk factors included in the PFS prediction model were AFP > 400 ng/ml (HR 1.35, 95%CI 1.20-1.53), ALBI >I (HR 1.40, 95%CI 1.25-1.56), NLR >3 (HR 1.34, 95%CI 1.15-1.55), ECOG performance status >0 (HR 1.69, 95%CI 1.34-2.15), Child-Pugh stage >A (HR 1.38, 95%CI 1.20-1.59), BCLC stage >B (HR 1.25, 95%CI 1.14-1.37), tumor number >1 (HR 1.26, 95%CI 1.08-1.47), and vascular invasion (HR 1.34, 95%CI 1.11-1.62). The β coefficients were 0.30, 0.34, 0.29, 0.53, 0.32, 0.22, 0.23, and 0.29, respectively. The study number of risk factors, sample size, pooled HR and 95%CI, β coefficient and risk score included in the risk prediction model for OS and PFS are detailed in **Table 1**.

**Table 1:** The β- coefficient and score of risk factors for prediction model of OS and PFS in HCC patients accepted ICI treatment.

Risk factor	Pooled HR	95%CI	β- coefficient	Score
The β- coefficient and score for OS prediction model				
AFP				
≤400 ng/ml	Reference	–	–	0
>400 ng/ml	1.51	1.37-1.66	0.412	4
ALBI				
≤I	Reference	–	–	0
>I	2.22	1.95-2.53	0.798	8
NLR				
≤3	Reference	–	–	0
>3	1.41	1.19-1.68	0.344	3.5
ECOG performance status				
≤0	Reference	–	–	0
>0 (Asian)	2.68	2.02-3.56	0.986	10
>0 (Non-Asian)	1.54	1.24-1.91	0.432	4.5
Child-Pugh stage				
≤A	Reference	–	–	0
>A	2.03	1.62-2.53	0.708	7
BCLC stage				
≤B	Reference	–	–	0
>B	1.40	1.23-1.58	0.336	3.5
Tumor number				
≤1	Reference	–	–	0
>1	1.63	1.14-2.34	0.489	5
Vascular invasion				
NO	Reference	–	–	0
YES	1.56	1.35-1.80	0.445	4.5
Concurrent therapy				
NO	Reference	–	–	0
YES	0.55	0.45-0.67	-0.598	-6
The β- coefficient and score for PFS prediction model				
AFP				
≤400 ng/ml	Reference	–	–	0
>400 ng/ml	1.35	1.20-1.53	0.300	3
ALBI				
≤I	Reference	–	–	0
>I	1.4	1.25-1.56	0.336	3.5
NLR				
≤3	Reference	–	–	0
>3	1.34	1.15-1.55	0.293	3
ECOG performance status				
≤0	Reference	–	–	0
>0	1.69	1.34-2.15	0.525	5
Child-Pugh stage				
≤A	Reference	–	–	0
>A	1.38	1.20-1.59	0.322	3
BCLC stage				
≤B	Reference	–	–	0
>B	1.25	1.14-1.37	0.223	2
Tumor number				
≤1	Reference	–	–	0
>1	1.26	1.08-1.47	0.231	2.5
Vascular invasion				
NO	Reference	–	–	0
YES	1.34	1.11-1.62	0.293	3

OS, overall survival; PFS, progression-free survival; ICI, immune checkpoint inhibitors; AFP, alpha-fetoprotein; ALBI, albumin-bilirubin score; NLR, neutrophil-to-lymphocyte ratio; ECOG, Eastern Cooperative Oncology Group.

### 3.3 Baseline characteristics of the 2 validation cohorts

Validation cohort 1 included 105 HCC patients treated with Atezolizumab. The patients had a mean age of 73.7 years, and 21% were female. The median follow-up was 6.4 months. In validation cohort 2, there were 99 HCC patients, with a mean patient age of 56.7 years, and 17% were female. The median follow-up was 12.2 months. Of these patients in validation cohort 2, 40 (40.4%) received Atezolizumab, 23 (23.2%) received Camrelizumab, 23 (23.2%) received Sintilimab, 7 (7.1%) received Tislelizumab, 4 (4.0%) received Toripalimab, 1 (1.0%) received Nivolumab, 1 (1.0%) received Penpulimab. The two cohorts totaled 204 patients, of whom 64 (31.4%) had AFP>400 ng/ml, 115 (56.4%) had ALBI>I, 81 (39.7%) had NLR>3, 32 (15.7%) had NLR>5, 49 (24.0%) had ECOG performance status>0, 40 (19.6%) had Child-Pugh stage>A, 115 (56.4%) had BCLC stage>B, 43 (21.1%) had vascular invasion, and 197 (96.6%) received concurrent therapy. The detailed baseline characteristics for both cohorts are presented in **Supplementary Table 6.**

4. Validation of the prediction models for OS and PFS in the 2 validation cohorts

**Figure 3:**
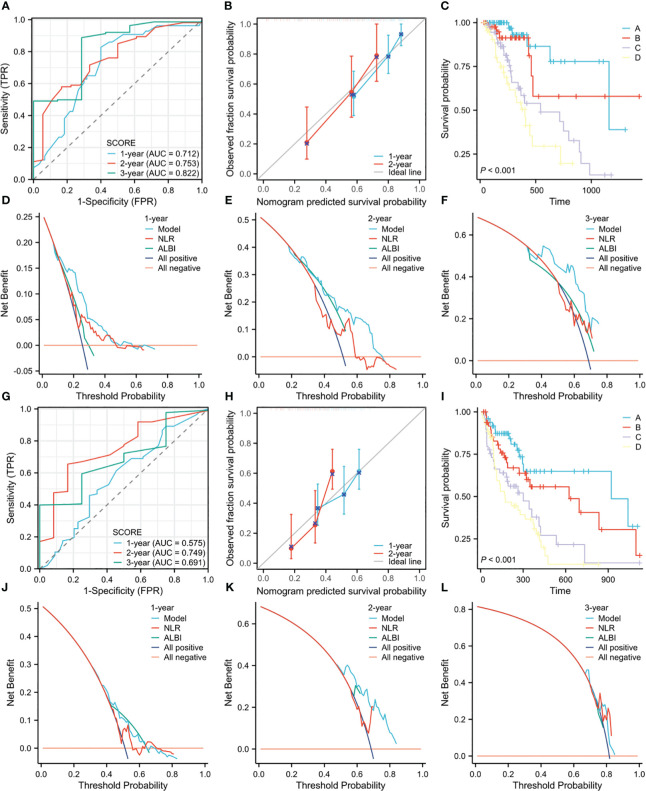
**(A)** The ROC curves for OS prediction model in the pooled cohort. **(B)** The calibration curves for the OS prediction model in the pooled cohort. **(C)** The KM curves of OS for the four risk groups in the pooled cohort. **(D–F)**. The 1-year, 2-year, and 3-year DCA curves for the OS prediction model in the pooled cohort. **(G)** The ROC curves for PFS prediction model in the pooled cohort. **(H)** The calibration curves for the PFS prediction model in the pooled cohort. **(I)** The KM curves of PFS for the four risk groups in the pooled cohort. **(J–L)**. The 1-year, 2-year, and 3-year DCA curves for the PFS prediction model in the pooled cohort.

In the validation cohort 1, the 0.3-, 0.6-, and 1-year area under the curves (AUC) of the model predicting OS were 0.775, 0.875 and 0.880, respectively (**Supplementary Figure 10A**), and the 0.3-, 0.6- and 1-year AUC of the model predicting PFS were 0.672, 0.703 and 0.750, respectively (**Supplementary Figure 10B**), indicating good prediction accuracy for both models. Furthermore, the calibration curves showed good agreement between the predicted and observed values of the OS and PFS models (**Supplementary Figures 10C, D**). In addition, there were significant differences in OS and PFS prognosis among the different risk subgroups (**Supplementary Figures 10E, F**). In validation cohort 2, the 1-, 2-, and 3-year AUC of the model predicting OS were 0.738, 0.780, and 0.838, respectively (**Supplementary Figure 11A**), and the 1-, 2-, and 3-year AUC of the model predicting PFS were 0.676, 0.829, and 0.757, respectively (**Supplementary Figure 11B)**, and the calibration curves also demonstrated that the predicted values of the models were consistent with the observed values (**Supplementary Figures 11C, D**). At the same time, the four risk subgroups divided by the model also showed significant differences in OS and PFS prognosis (**Supplementary Figures 11E, F)**. We noted that although the differences between the two validation cohorts were large, the OS and PFS prediction models showed good performance in both cohorts. Moreover, in the combined cohort of 204 patients, the 1-, 2-, and 3-year AUC of the model for predicting OS were 0.712, 0.753, and 0.822, respectively (**Figure 3A**), and the 1-, 2-, and 3-year AUC of the model for predicting PFS were 0.575, 0.749 and 0.691, respectively (**Figure 3G**). The calibration curves showed that the prediction curves of the model were close to the ideal curves (**Figures 3B, H**), the KM curves showed that there were differences in OS and PFS prognosis of patients in different risk subgroups (**Figures 3C, I**), and the DCA curves demonstrated that the models could provide net benefit for patients than ALBI and NLR, which are both significant prognostic variables in previous studies (24–26) (**Figures 3D–F, J–L**). Based on the validation results, we developed two Web calculators that can predict the prognosis of ICI-treated HCC patients. The web calculators for OS and PFS can be accessed through following links: https://icipredictionmodel.shinyapps.io/OS-Prediction-app/and https://icipredictionmodel.shinyapps.io/PFS-Prediction-app/.

## Discussion

This study conducted a systematic review and meta-analysis of 7649 HCC patients treated with ICI in 47 studies, and finally identified 8 OS-related risk factors and 9 PFS-related risk factors from 18 risk factors. According to the pooled HR values of these risk factors, OS and PFS prediction models were constructed respectively. Furthermore, 2 cohorts of ICI-treated HCC patients from Japan and China (totaling 105 and 99 patients, respectively) and the pooled cohort were used to validate the predictive value of these models. Through the above validation, we fully demonstrate the predictive accuracy and suitability of these models for further clinical application.

AFP is a widely used serum biomarker in clinical management of HCC patients (27). Several studies have shown that elevated AFP levels are associated with poor prognosis among HCC patients at various developmental stages or receiving different treatment methods (28–31). While many studies had examined the correlation between AFP and the prognosis of HCC patients receiving ICI treatment, their conclusions exhibit significant variability (14, 32, 33). Our meta-analysis affirmed that raised AFP levels were associated with worse OS and PFS outcomes, consistent with results from a recent meta-analysis conducted by Zhang et al (34). Additionally, studies have shown that AFP could inhibit T-lymphocyte proliferation and cytotoxicity, interfere with natural killer cell function and dendritic cell differentiation, promote an immunosuppressive tumor microenvironment, and thus reduce the efficacy of ICI treatments (35). In addition, several new studies have suggested that AFP is related to the activation of tumor vascular endothelial growth factor (VEGF) pathway, which might hinder anti-tumor immune responses by influencing the function and infiltration of immune cells (27, 36, 37).

HCC is an inflammation-driven malignancy, as most HCC are often accompanied by chronic inflammation (18, 38). Considerable evidence supported that inflammatory processes contribute to cancer initiation, promotion, progression, and invasion (17, 39). At the same time, more and more studies have found that inflammatory process is related to the efficacy of immunotherapy (30, 40, 41). Therefore, some biomarkers related to inflammation may be ideal for predicting the prognosis of immunotherapy. Among them, NLR and PLR are two easy to obtain biomarkers that can reflect the balance between inflammatory state and anti-tumor immune state of patients. The former is obtained from the ratio of peripheral blood neutrophils to lymphocytes, while the latter is obtained from the ratio of peripheral blood platelets to lymphocytes. Liu et al. conducted a meta-analysis to demonstrate the prognostic value of NLR and PLR in HCC patients treated with sorafenib (42). For HCC patients receiving ICI treatment, many studies reported a strong predictive role of NLR and PLR for prognosis, but also some studies reported negative results (20, 43, 44). A recent meta-analysis found that both NLR and PLR to be associated with the prognosis of HCC patients receiving ICI treatment, and NLR was additionally related to objective response rate and disease control rate (34). In our study, NLR was associated with OS and PFS, while PLR was associated with PFS. However, the pooled analysis between PLR and OS was not significant, which may be caused by too few studies (only two studies).

The current researches on the prognostic risk factors of HCC patients receiving ICI treatment mainly have primarily relied on cohort studies involving sample sizes spanning tens to hundreds of patients. However, due to differences among the patient populations enrolled in these studies, there is substantial variability in the resulting risk factor analyses. Systematic review and meta-analysis of homogeneous studies can expand the sample size, enhance the statistical power and precision of estimated effect sizes, and improve the objectivity and reliability of research findings (45). Accordingly, we developed an OS and PFS risk prediction model for HCC patients receiving ICI treatment based on our meta-analysis results. Our model facilitates risk assessment by assigning values to different risk factors, enabling rapid evaluation of patients’ probability of recurrence or mortality. Some previous studies had developed such as the hepatocellular carcinoma modified Gustave Roussy Immune Score (HCC-GRIm) or CRP and AFP in ImmunoTherapY (CRAFITY) score for risk stratification of HCC patients receiving ICI treatment, so as to facilitate clinicians to intervene on patients (26, 46). However, these scoring systems have limitations in failing to take into account tumor-related features such as number and size or other clinical interventions such as concurrent therapy. In contrast, our model was constructed taking these factors into account. The results of this study showed that the 1-year and 2-year AUC of the OS model were 0.745 and 0.780 in cohort 2, and 0.663 and 0.743 in the pooled cohort. In the CRAFITY score developed by Scheiner et al., the 1-year and 2-year AUC for predicting OS were 0.71 and 0.69 in the training set, and 0.71 and 0.69 in the validation set (46). Thus, our study demonstrates that our model’s predictive ability is comparable to that of the well-established CRAFITY scoring system.

Notably, while ICIs are gradually replacing sorafenib as the first-line treatment for HCC, there are also effective second-line immunotherapy options being clinically utilized, such as nivolumab and pembrolizumab (47, 48). A network meta-analysis conducted by Solimando, A G demonstrated that pembrolizumab, as a second-line treatment, significantly prolongs PFS compared to placebo (6). Unfortunately, due to limitations in the number of original studies and the sample size of validation cohorts, we were unable to further study the efficacy of first-line and second-line treatments. Future research can focus on this aspect to explore it in more depth.

There are still some limitations in this study. Our model was constructed based on the results of meta-analysis, therefore some methodological limitations that could potentially affect the study results, some of which are unavoidable, such as (1) Language bias: Due to limitations in resources and time, our meta-analysis relied on original literature from four English databases. This reliance may introduce a certain degree of language bias that could potentially impact the study results (2). Heterogeneity: Most of the included studies were retrospective cohort studies, and each study had different design and included patients. Therefore, even though we evaluated heterogeneity using Cochran’s Q test and Higgins inconsistency index, and flexibly used both random-effects and fixed-effects models to calculate meta-analytic effect size, there remains an impact of heterogeneity on the results that cannot be entirely eliminated (3). Publication bias: Although we performed Begg’s and Egger’s tests to assess publication bias, these methods have limitations and do not provide complete assurance against the presence of publication bias. In future research, more effort can be devoted to including unpublished studies to reduce the impact of publication bias resulting from unpublished negative results (4). Model validation: Due to limitations in the number of hospitals involved and ethical review, the sample size in both validation cohorts is relatively small, and there may be potential issues such as limited model generalizability (5). Generalizability: Although we successfully established a personalized prediction model of OS based on regional differences, the model for non-Asian populations cannot be well-validated due to the lack of a validation cohort for this group, and the inclusion of primarily Asian region studies and validation patients may limit the generalizability of our predictive models. Additionally, we excluded patients with ICC and cHCC-CC in the methodology section, which means this model may not be applicable to other subtypes of liver cancer.

In conclusion, our study has constructed OS and PFS prediction models based on meta-analysis results, which were then successfully validated in two independent cohorts. Because the model’s selected indicators are simple to obtain in a clinical setting, it possesses high practicality and can help pinpoint treatment gaps needing targeted interventions. In order to further verify the robustness of the models, prospective validation in large clinical studies is required in the future.

## Data availability statement

The raw data supporting the conclusions of this article will be made available by the authors, without undue reservation.

## Author contributions

DM: Conceptualization, Methodology, Software, Investigation, Formal Analysis, Writing - Original Draft. ML: Data Curation, Software, Investigation, Formal Analysis, Writing - Original Draft. XZ: Visualization, Investigation, Writing - Original Draft. XL: Resources, Visualization, Supervision. YL: Methodology, Funding Acquisition, Visualization, Writing - Review & Editing. BJ: Conceptualization, Funding Acquisition, Resources, Supervision, Writing - Review & Editing. All authors contributed to the article and approved the submitted version.
