# Supplementary material for: Development and validation of prognostic risk prediction models for hepatocellular carcinoma patients treated with immune checkpoint inhibitors based on a systematic review and meta-analysis of 47 cohorts
Source: Front Immunol. 2023 Jul 14;14:1215745. doi: 10.3389/fimmu.2023.1215745 (PMC10380940; doi:10.3389/fimmu.2023.1215745)
Supplement: Supplementary file 1 [file DataSheet_1.pdf]

Supplementary file

1. Literature search strategy

Pubmed (119)

((((((("Carcinoma, Hepatocellular"[Mesh]) OR (hepatocellular carcinoma)) OR (liver cell carcinoma)) OR (hepatoma)) OR (HCC)) AND ((((((((((((("Immune Checkpoint Inhibitors"[Mesh]) OR (CTLA 4 Inhibitor)) OR (PD-L1 Inhibitor)) OR (PD-1 Inhibitor)) OR (PD-1 antibody)) OR (PD-L1 antibody)) OR (Anti-PD-1)) OR (Anti-PD-L1)) OR (Atezolizumab)) OR (Pembrolizumab)) OR (Nivolumab)) OR (Ipilimumab)) OR (Sintilimab)) OR (Tislelizumab)) OR (Camrelizumab))) AND (((((((((((((((((((((((((((((("Risk Factors"[Mesh]) OR (BMI)) OR (body mass index)) OR (Child Pugh)) OR (BCLC stage)) OR (tumor size)) OR (tumor number)) OR (vascular invasion)) OR (metastasis)) OR (AFP)) OR (alpha-fetoprotein)) OR (bilirubin)) OR (CHO)) OR (cholesterol)) OR (CRP)) OR (C-reactive protein)) OR (ALB)) OR (albumin)) OR (LDH)) OR (lactate dehydrogenase)) OR (NLR)) OR (neutrophil-to-lymphocyte ratio)) OR (PLR)) OR (platelet-to-lymphocyte ratio)) OR (SII)) OR (systemic immune-inflammation index)) OR (LMR)) OR (lymphocyte-to-monocyte ratio)) OR (PNI)) OR (prognostic nutritional index)) OR (HBV)) OR (Hepatitis B virus)) OR (CAR)) OR (C-reactive protein-to-albumin ratio))) AND (("Survival"[Mesh]) OR "Prognosis"[Mesh])) AND ("Cohort Studies"[Mesh])

Web of science (156)

1 TS=(carcinoma, hepatocellular) OR TS=(hepatocellular carcinoma) OR TS=(liver cell carcinoma) OR TS=(hepatoma) OR TS=(HCC) (370739)

2 TS=(Immune Checkpoint Inhibitors) OR TI=("CTLA 4 Inhibitor" OR "PD-L1 Inhibitor" OR "PD-1 Inhibitor" OR "PD-1 antibody" OR "PD-L1 antibody" OR "Anti-PD-1" OR "Anti-PD-L1" OR "Atezolizumab" OR "Pembrolizumab" OR "Nivolumab" OR "Ipilimumab" OR "Sintilimab" OR "Tislelizumab" OR "Camrelizumab") OR AB=("CTLA 4 Inhibitor" OR "PD-L1 Inhibitor" OR "PD-1 Inhibitor" OR "PD-1 antibody" OR "PD-L1 antibody" OR "Anti-PD-1" OR "Anti-PD-L1" OR "Atezolizumab" OR "Pembrolizumab" OR "Nivolumab" OR "Ipilimumab" OR "Sintilimab" OR "Tislelizumab" OR "Camrelizumab") (70108)

3 TS=(Risk Factors) OR TI=("BMI" OR "body mass index" OR "Child Pugh" OR "BCLC stage" OR "tumor size" OR "tumor number" OR "vascular invasion" OR "metastasis" OR "AFP" OR "alpha-fetoprotein" OR "bilirubin" OR "CHO" OR "cholesterol" OR "CRP"OR "C-reactive protein" OR "ALB" OR "albumin" OR "LDH" OR "lactate dehydrogenase"OR "NLR" OR "neutrophil-to-lymphocyte ratio" OR "PLR" OR "platelet-to-lymphocyte ratio" OR "SII" OR "systemic immune-inflammation index" OR "LMR" OR "lymphocyte-to-monocyte ratio" OR "PNI" OR "prognostic nutritional index"OR "HBV" OR "Hepatitis B virus" OR "CAR" OR "C-reactive protein-to-albumin ratio") OR AB=("BMI" OR "body mass index" OR "Child Pugh" OR "BCLC stage" OR "tumor size" OR "tumor number" OR "vascular invasion" OR "metastasis" OR "AFP" OR "alpha-fetoprotein" OR "bilirubin" OR "CHO" OR "cholesterol" OR "CRP"OR "C-reactive protein" OR "ALB" OR "albumin" OR "LDH" OR "lactate dehydrogenase"OR "NLR" OR "neutrophil-to-lymphocyte ratio" OR "PLR" OR "platelet-to-lymphocyte ratio" OR "SII" OR "systemic immune-inflammation index" OR "LMR" OR "lymphocyte-to-monocyte ratio" OR "PNI" OR "prognostic nutritional index"OR "HBV" OR "Hepatitis B virus" OR "CAR" OR "C-reactive protein-to-albumin ratio") (5314233)

4 TS=(Survival) OR TS=(Prognosis) (3103346)

5 TS=(Cohort Studies) (1070167)

6 #1 AND #2 AND #3 AND #4 AND #5 (156)

Embase (193)

1 'liver cell carcinoma'/exp OR 'hepatocellular carcinoma':ti,ab,kw OR 'liver cell carcinoma':ti,ab,kw OR hepatoma:ti,ab,kw OR hcc:ti,ab,kw (254078)

2 'immune checkpoint inhibitor'/exp OR 'ctla 4 inhibitor':ti,ab,kw OR 'pd-l1 inhibitor':ti,ab,kw OR 'pd-1 inhibitor':ti,ab,kw OR 'pd-1 antibody':ti,ab,kw OR 'pd-l1 antibody':ti,ab,kw OR 'anti pd 1':ti,ab,kw OR 'anti pd l1':ti,ab,kw OR atezolizumab:ti,ab,kw OR pembrolizumab:ti,ab,kw OR nivolumab:ti,ab,kw OR ipilimumab:ti,ab,kw OR sintilimab:ti,ab,kw OR tislelizumab:ti,ab,kw OR

camrelizumab:ti,ab,kw (70089)
3 'risk factor'/exp OR bmi:ti,ab,kw OR 'body mass index':ti,ab,kw OR 'child pugh':ti,ab,kw OR 'bclc stage':ti,ab,kw OR 'tumor size':ti,ab,kw OR 'tumor number':ti,ab,kw OR 'vascular invasion':ti,ab,kw OR metastasis:ti,ab,kw OR afp:ti,ab,kw OR 'alpha fetoprotein':ti,ab,kw OR bilirubin:ti,ab,kw OR cho:ti,ab,kw OR cholesterol:ti,ab,kw OR crp:ti,ab,kw OR 'c-reactive protein':ti,ab,kw OR alb:ti,ab,kw OR albumin:ti,ab,kw OR ldh:ti,ab,kw OR 'lactate dehydrogenase':ti,ab,kw OR nlr:ti,ab,kw OR 'neutrophil-to-lymphocyte ratio':ti,ab,kw OR plr:ti,ab,kw OR 'platelet-to-lymphocyte ratio':ti,ab,kw OR sii:ti,ab,kw OR 'systemic immune-inflammation index':ti,ab,kw OR lmr:ti,ab,kw OR 'lymphocyte-to-monocyte ratio':ti,ab,kw OR pni:ti,ab,kw OR 'prognostic nutritional index':ti,ab,kw OR hbv:ti,ab,kw OR 'hepatitis b virus':ti,ab,kw OR car:ti,ab,kw OR 'c-reactive protein-to-albumin ratio':ti,ab,kw (3210850)
4 'survival'/exp OR 'prognosis'/exp (1958573)
5 'cohort analysis'/exp (971545)
4 #1 AND #2 AND #3 AND #4 AND #5 (193)

Cochrane Library literature databases (9)
#1 MeSH descriptor: [Carcinoma, Hepatocellular] explode all trees 2357
#2 (hepatocellular carcinoma):ti,ab,kw OR (liver cell carcinoma):ti,ab,kw OR (hepatoma):ti,ab,kw OR (HCC):ti,ab,kw (Word variations have been searched) 7850
#3 MeSH descriptor: [Immune Checkpoint Inhibitors] explode all trees 180
#4 (CTLA 4 Inhibitor OR PD-L1 Inhibitor OR PD-1 Inhibitor OR PD-1 antibody OR PD-L1 antibody OR Anti-PD-1 OR Anti-PD-L1 OR Atezolizumab OR Pembrolizumab OR Nivolumab OR Ipilimumab OR Sintilimab OR Tislelizumab OR Camrelizumab):ti,ab,kw (Word variations have been searched) 8429
#5 MeSH descriptor: [Risk Factors] explode all trees 32969
#6 (BMI OR body mass index OR Child Pugh OR BCLC stage OR tumor size OR tumor number OR vascular invasion OR metastasis OR AFP OR alpha-fetoprotein OR bilirubin OR CHO OR cholesterol OR CRP OR C-reactive protein OR ALB OR albumin OR LDH OR lactate dehydrogenase OR NLR OR neutrophil-to-lymphocyte ratio OR PLR OR platelet-to-lymphocyte ratio OR SII OR systemic immune-inflammation index OR LMR OR lymphocyte-to-monocyte ratio OR PNI OR prognostic nutritional index OR HBV OR Hepatitis B virus OR CAR OR C-reactive protein-to-albumin ratio):ti,ab,kw (Word variations have been searched)217346
#7 MeSH descriptor: [Survival] explode all trees 165
#8 MeSH descriptor: [Prognosis] explode all trees 193305
#9 MeSH descriptor: [Cohort Studies] explode all trees 181684
#10 #1 OR #2 7850
#11 #3 OR #4 8468
#12 #5 OR #6 242452
#13 #7 OR #8 193373
#14 #10 AND #11 AND #12 AND #13 AND #9 9

2. Supplementary Table

Supplementary Table 1. Baseline characteristics of the 47 studies included in the systematic review and meta-analysis.

Author	Country	Year	Age (years, median / mean)	Study design	Sample size	ICI type	Follow-up time (months)	Sample for OS and PFS	NOS score
Wei Teng[1]	China	2021	61.4	R	90	PD-1 inhibitors	8.7	Sex, AFP, Age, ALBI, BCLC stage, Viral infection, IrAE, Extrahepatic metastasis	8
Mengchao An[2]	China	2022	54	R	217	PD-1 inhibitors	NA	Sex, AFP, Age, ALBI, BCLC stage, Child-Pugh stage, ECOG performance status, Liver cirrhosis, Vascular invasion, Concurrent therapy, Tumor size, Tumor number, Extrahepatic metastasis	7
Shixue Chen[3]	China	2020	56	R	108	PD-1 inhibitors	12.4	Sex, AFP, BCLC stage, Child-Pugh stage, ECOG performance status, Viral infection, Vascular invasion, Previous treatment, Concurrent therapy	8
Tiago de Castro[4]	Germany	2022	68.7	R	147	PD-L1 inhibitors	6.2	ALBI, BCLC stage, ECOG performance status, Viral infection, Previous treatment	6
Claudia Campani[5]	France	2022	63.5	R	75	PD-L1 inhibitors	15.6	Sex, ALBI, BCLC stage, Viral infection, Liver cirrhosis, Vascular invasion, Tumor number, Extrahepatic metastasis	6
Rui Huang[6]	China	2022	54.5	R	110	PD-1 inhibitors	NA	Sex, AFP, Age, BCLC stage, Child-Pugh stage, ECOG performance status, NLR, PLR, Vascular invasion	8
Yiyang Zhang[7]	China	2022	49.4	R	101	PD-1 inhibitors	7.7	Sex, AFP, Age, Child-Pugh stage, ECOG performance status, Viral infection, Tumor size, Tumor number, Extrahepatic metastasis	7
Yanjun Shen[8]	China	2021	58	R	57	PD-1 inhibitors	9.8	Sex, AFP, Age, BCLC stage, Child-Pugh stage, Liver cirrhosis, Vascular invasion, Tumor size, Tumor number, Extrahepatic metastasis	8
Junlin Yao[9]	China	2022	58	R	136	PD-1 inhibitors	14.2	Sex, AFP, Age, BCLC stage, Child-Pugh stage, ECOG performance status, Viral infection, Liver cirrhosis, Previous treatment, Extrahepatic metastasis	7
Fabian Finkelmeier[10]	Germany	2019	65	R	34	PD-1 inhibitors	3.3	Sex, Age, BCLC stage, Child-Pugh stage, ECOG performance status, NLR, Previous treatment	8
Lei Xu[11]	China	2021	51.8	R	74	PD-1 inhibitors	NA	AFP, Age, Child-Pugh stage, Previous treatment, Concurrent therapy	7
Hsin-Yu Kuo[12]	China	2020	58	R	42	PD-1 inhibitors	4.6	Sex, AFP, Age, Child-Pugh stage, ECOG performance status, Vascular invasion, Previous treatment, Concurrent therapy, Extrahepatic metastasis	6
Kennedy	Singapor	2021	69	R	168	NA	25.1	IrAE	7

Yao Yi Ng[13]	e								
Mathew Vithayathil[14]	UK	2022	68.4	R	191	PD-L1 inhibitors	NA	AFP, Age, BCLC stage, Vascular invasion, Tumor size, Extrahepatic metastasis	8
Nalee Kim[15]	Korea	2020	61.3	R	102	PD-1 inhibitors	21.9	Sex, Age, ALBI, ECOG performance status, Viral infection, Previous treatment, Extrahepatic metastasis	7
Xiaoyun Hu[16]	China	2022	52.5	R	70	PD-1 inhibitors	NA	Sex, AFP, BCLC stage, Child-Pugh stage, Vascular invasion, Tumor size, Tumor number, Extrahepatic metastasis	7
Xu Yang[17]	China	2022	55	R	378	PD-1 inhibitors	10.4	AFP, BCLC stage, ECOG performance status, Vascular invasion, Concurrent therapy	8
Chi- Jung Wu[18]	China	2022	63	P	71	PD-1 inhibitors	9.3	Sex, AFP, Age, ALBI, BCLC stage, Child-Pugh stage, ECOG performance status, NLR, Viral infection, Vascular invasion, Previous treatment, Tumor number, Extrahepatic metastasis	8
Yue Linda Wu[19]	USA	2022	66	R	296	PD-L1 inhibitors	9.93	Sex, AFP, ALBI, BCLC stage, ECOG performance status, NLR, Viral infection, Liver cirrhosis, Vascular invasion, Previous treatment, Extrahepatic metastasis	9
Toshifumi Tada[20]	Japan	2022	74	R	421	PD-L1 inhibitors	8.7	Sex, AFP, Age, BCLC stage, NLR, PLR, Viral infection	7
Sirish Dharmapuri[21]	USA	2020	67	R	103	PD-1 inhibitors	17	BCLC stage, Child-Pugh stage	8
Pei-Chang Lee[22]	China	2020	65.5	R	95	PD-1 inhibitors	NA	Sex, AFP, Age, ALBI, BCLC stage, Child-Pugh stage, NLR, IrAE, Vascular invasion, Previous treatment, Concurrent therapy, Tumor size, Tumor number, Extrahepatic metastasis	7
Wei-Fan Hsu[23]	China	2020	63.4	R	87	PD-1 inhibitors	NA	Sex, AFP, ALBI, Child-Pugh stage, NLR, Viral infection, IrAE, Vascular invasion, Previous treatment, Concurrent therapy, Tumor number, Extrahepatic metastasis	7
Bernhard Scheiner[24]	Austria	2021	66.2	R	190	Mixed	15.6	AFP, Child-Pugh stage, ECOG performance status, Viral infection, Vascular invasion, Extrahepatic metastasis	9
Xiaomi Li[25]	China	2022	59	R	258	PD-1 inhibitors	NA	ECOG performance status, Concurrent therapy, Extrahepatic metastasis	7
Zilong Zhang[26]	China	2022	55	R	101	PD-1 inhibitors	11.1	BCLC stage, Child-Pugh stage, Concurrent therapy, Tumor number, Extrahepatic	7

								metastasis	
Pil Soo Sung[27]	Korea	2020	57	R	33	PD-1 inhibitors	12.5	Sex, AFP, Age, ALBI, Vascular invasion, Tumor size	7
Xuqi Sun[28]	China	2021	51	R	235	PD-1 inhibitors	NA	Sex, AFP, Age, ALBI, Vascular invasion, Extrahepatic metastasis	8
Mara Persano[29]	Italy	2023	72	R	773	PD-L1 inhibitors	NA	Sex, AFP, Age, BCLC stage, Child-Pugh stage, NLR, Viral infection, Previous treatment	9
Guosheng Yuan[30]	China	2020	48.7	R	63	PD-1 inhibitors	12.6	Sex, AFP, Child-Pugh stage, Tumor size, Tumor number	7
Bang-Bin Chen[31]	China	2023	59	R	111	Mixed	NA	Sex, AFP, BCLC stage, Vascular invasion, Tumor number, Extrahepatic metastasis	8
Takeshi Hatanaka[32]	Japan	2022	74	R	405	PD-L1 inhibitors	NA	Sex, AFP, ALBI, BCLC stage, Viral infection	7
Muhammad O Awiwi[33]	USA	2022	66	R	55	PD-L1 inhibitors	7.9	ALBI, PLR, Vascular invasion	8
Jing-Houng Wang[34]	China	2022	62	R	48	PD-L1 inhibitors	9.5	AFP, PLR, Previous treatment	8
Jin-Tao Huang[35]	China	2022	NA	R	64	PD-1 inhibitors	23	Sex, AFP, Child-Pugh stage, ECOG performance status, Viral infection, Vascular invasion, Concurrent therapy, Tumor number, Extrahepatic metastasis	7
David J. Pinato[36]	UK	2021	64	P	357	Mixed	15.3	Sex, AFP, BCLC stage, Child-Pugh stage, ECOG performance status, Viral infection, Liver cirrhosis	9
Ka Shing Cheung[37]	China	2021	61	R	395	Mixed	16.5	Sex, AFP, Child-Pugh stage, Liver cirrhosis, Previous treatment	7
Hironori Ochi[38]	Japan	2022	NA	R	242	PD-L1 inhibitors	Atezolizumab	Sex, AFP, Age, ALBI, BCLC stage, ECOG performance status, NLR, Viral infection, Vascular invasion, Extrahepatic metastasis	7
Sara Lewis[39]	USA	2021	61.5	R	58	PD-1 Inhibitors	NA	Previous treatment	6
Yuka Hayakawa[40]	Japan	2021	73	R	46	PD-L1 inhibitors	7.37	BCLC stage, Viral infection, Extrahepatic metastasis	8
Ming-Hao Xu[41]	China	2023	57	R	210	PD-1 inhibitors	19.8	Sex, AFP, Age, ALBI, BCLC stage, Child-Pugh stage, ECOG performance status, Viral infection, Vascular invasion, Tumor size, Extrahepatic metastasis	8
Won-Mook Choi[42]	Korea	2020	NA	R	203	PD-1 Inhibitors	5.6	Sex, AFP, ALBI, BCLC stage, Child-Pugh stage, ECOG performance status, Viral infection, Vascular invasion, Extrahepatic metastasis	7

Yan-Jun Xiang[43]	China	2022	NA	R	76	PD-1 Inhibitor s	NA	Sex, AFP, Age, ALBI, Viral infection, Liver cirrhosis, Tumor number	7
Takuya Sho[44]	Japan	2022	72	R	115	PD-L1 inhibitors	6.8	ALBI, BCLC stage, Child-Pugh stage, Viral infection, Vascular invasion	7
Takeshi Hatanaka[45]	Japan	2022	73	R	297	PD-L1 inhibitors	NA	Sex, AFP, Age, BCLC stage, Viral infection	9
Dezuo Dong[46]	China	2022	57	R	38	PD-1 inhibitors	16.5	Sex, AFP, BCLC stage, ECOG performance status, NLR, Liver cirrhosis, Vascular invasion, Previous treatment, Extrahepatic metastasis	7
Yan-Jun Xiang[47]	China	2022	NA	R	103	PD-1 inhibitors	11.4	Sex, AFP, Age, ALBI, Viral infection, Liver cirrhosis, Concurrent therapy, Tumor size	8

Previous treatment included surgery, locoregional treatment (TACE, radiofrequency ablation, radiation therapy), and systemic therapy (antiangiogenic therapy, target therapy).

Concurrent therapy included locoregional treatment (TACE, radiofrequency ablation, radiation therapy), and systemic therapy (antiangiogenic therapy, target therapy).

Supplementary Table 2. Quality assessment of the 47 studies by checklist (based on Newcastle-Ottawa Scale)

Study	Selection				Comparability	Outcome			Total Score
	I	II	III	IV	V	VI	VII	VIII	
Wei Teng 2021	★	★	★	★	★★	★	☆	★	8
Mengchao An 2022	☆	★	★	★	★☆	★	★	★	7
Shixue Chen 2020	★	★	★	★	★★	★	☆	★	8
Tiago de Castro 2022	☆	★	★	★	★☆	★	☆	★	6
Claudia Campani 2022	★	☆	★	★	★☆	★	☆	★	6
Rui Huang 2022	★	★	★	★	★★	★	☆	★	8
Yiyang Zhang 2022	★	☆	★	★	★☆	★	★	★	7
Yanjun Shen 2021	★	★	★	★	★☆	★	★	★	8
Junlin Yao 2022	☆	★	★	★	★☆	★	★	★	7
Fabian Finkelmeier 2019	★	★	★	★	★☆	★	★	★	8
Lei Xu 2021	★	☆	★	★	★★	★	★	☆	7
Hsin-Yu Kuo 2020	☆	★	★	★	★☆	★	★	☆	6
Kennedy Yao Yi Ng 2022	☆	★	★	★	★☆	★	★	★	7
Mathew Vithayathil 2022	★	★	★	★	★☆	★	★	★	8
Nalee Kim 2020	☆	★	★	★	★☆	★	★	★	7
Xiaoyun Hu 2022	★	★	★	★	★★	★	☆	☆	7
Xu Yang 2022	★	★	★	★	★☆	★	★	★	8
Chi-Jung Wu 2022	★	★	★	★	★☆	★	★	★	8
Yue Linda Wu 2022	★	★	★	★	★★	★	★	★	9
Toshifumi Tada 2022	★	★	★	★	★☆	★	☆	★	7

Sirish Dharmapuri 2022	★	★	★	★	★☆	★	★	★	8
Pei-Chang Lee 2020	★	☆	★	★	★★	★	☆	★	7
Wei-Fan Hsu 2020	☆	★	★	★	★☆	★	★	★	7
Bernhard Scheiner 2021	★	★	★	★	★★	★	★	★	9
Xiaomi Li 2022	★	☆	★	★	★☆	★	★	★	7
Zilong Zhang 2022	☆	★	★	★	★☆	★	★	★	7
Pil Soo Sung 2020	★	★	★	★	★☆	★	☆	★	7
Xuqi Sun 2021	★	☆	★	★	★★	★	★	★	8
Mara Persano 2023	★	★	★	★	★★	★	★	★	9
Guosheng Yuan 2020	☆	★	★	★	★☆	★	★	★	7
Bang-Bin Chen 2022	★	☆	★	★	★★	★	★	★	8
Takeshi Hatanaka 2022	★	★	★	★	★☆	★	☆	★	7
Muhammad O Awiwi 2022	☆	★	★	★	★★	★	★	★	8
Jing-Houng Wang 2022	★	★	★	★	★★	★	☆	★	8
Jin-Tao Huang 2022	☆	★	★	★	★☆	★	★	★	7
David J. Pinato 2021	★	★	★	★	★★	★	★	★	9
Ka Shing Cheung 2021	☆	★	★	★	★☆	★	★	★	7
Hironori Ochi 2022	★	★	★	★	★☆	★	☆	★	7
Sara Lewis 2021	☆	☆	★	★	★★	★	☆	★	6
Takeshi Hatanaka 2022	☆	★	★	★	★★	★	☆	★	7
Yuka Hayakawa 2021	★	★	★	★	★☆	★	★	★	8
Ming-Hao Xu 2023	★	★	★	★	★★	★	☆	★	8

Won-Mook Choi 2020	☆	★	★	★	★☆	★	★	★	7
Yan-Jun Xiang 2022	☆	★	★	★	★★	★	☆	★	7
Takuya Sho 2022	★	★	★	★	★☆	★	☆	★	7
Takeshi Hatanaka 2022	★	★	★	★	★★	★	★	★	9
Dezuo Dong 2022	☆	★	★	★	★☆	★	★	★	7
Yan-Jun Xiang 2022	★	★	★	★	★☆	★	★	★	8

Abbreviations: I, Representativeness of the exposed cohort; II, Selection of the non-exposed cohort; III, Ascertainment of expsure; IV, Demonstration that outcome of interest was not present at start of study; V, Comparability of cohorts on the basis of the design or analysis; VI, Assessment of outcome; VII, Was follow-up long enough for outcomes to occur; VIII, Adequacy of follow up of cohorts.

★, Asterisk means that the study is satisfied the item; ☆, Asterisk means the opposite situation.

A score>6 was defined as high quality and ≤6 was defined as low quality.

Supplementary Table 3. Results of meta-analysis of risk factors of OS and PFS in HCC patients accepted ICI treatment.

Risk factors	Outcome	No. of articles	Sample size	Heterogeneity test		Effects model	Pooled results		
				I^2	P		HR	95%CI	P
Sex	OS	27	4828	0%	0.46	Fixed	0.89	0.79-1.00	0.05
	PFS	28	5428	30%	0.07	Random	1.01	0.89-1.15	0.90
AFP	OS	29	5915	29%	0.07	Random	1.51	1.37-1.66	<0.01
	PFS	27	5114	55%	<0.01	Random	1.35	1.20-1.53	<0.01
Age	OS	16	2817	41%	0.05	Random	1.04	0.89-1.21	0.65
	PFS	18	3357	27%	0.14	Fixed	1.00	0.97-1.02	0.8
ALBI	OS	15	2321	0%	0.64	Fixed	2.22	1.95-2.53	<0.01
	PFS	16	2582	19%	0.24	Fixed	1.40	1.25-1.56	<0.01
BCLC stage	OS	26	5094	3%	0.42	Fixed	1.40	1.23-1.58	<0.01
	PFS	23	4857	0%	0.96	Fixed	1.25	1.14-1.37	<0.01
Child-Pugh stage	OS	20	3416	48%	<0.01	Random	2.03	1.62-2.53	<0.01
	PFS	16	2731	0%	0.52	Fixed	1.38	1.20-1.59	<0.01
ECOG performance status	OS	18	2961	62%	<0.01	Random	2.26	1.77-2.88	<0.01
	PFS	15	2611	67%	<0.01	Random	1.69	1.34-2.15	<0.01
Viral infection	OS	18	4032	0%	0.65	Fixed	0.98	0.88-1.10	0.77
	PFS	21	4314	7%	0.36	Fixed	1.03	0.94-1.12	0.55
Liver cirrhosis	OS	8	1571	72%	<0.01	Random	1.41	0.89-2.24	0.15
	PFS	9	1355	41%	0.09	Random	1.07	0.85-1.33	0.57
Vascular invasion	OS	22	2981	29%	0.10	Random	1.56	1.35-1.80	<0.01
	PFS	18	2605	59%	<0.01	Random	1.34	1.11-1.62	<0.01
Tumor size	OS	7	866	56%	0.03	Random	1.37	0.96-1.96	0.09
	PFS	6	758	0%	0.93	Fixed	1.04	0.85-1.26	0.71
Tumor number	OS	10	941	54%	0.02	Random	1.63	1.14-2.34	<0.01
	PFS	9	865	27%	0.21	Fixed	1.26	1.08-1.47	<0.01
Extrahepatic metastasis	OS	21	2869	50%	<0.01	Random	1.14	0.96-1.35	0.12
	PFS	20	2530	49%	<0.01	Random	1.11	0.96-1.29	0.14
NLR	OS	10	2167	40%	0.09	Random	1.41	1.19-1.68	<0.01
	PFS	11	2074	20%	0.26	Fixed	1.34	1.15-1.55	<0.01
PLR	OS	2	406	67%	0.08	Random	1.62	0.56-4.65	0.37
	PFS	4	509	52.00%	0.10	Random	2.26	1.13-4.55	0.02
Previous treatment	OS	12	2201	42%	0.06	Random	0.93	0.79-1.10	0.42
	PFS	14	2014	36%	0.09	Random	0.95	0.84-1.07	0.39
IrAE	OS	4	440	70%	0.02	Random	0.76	0.42-1.35	0.35
	PFS	3	345	86%	<0.01	Random	0.79	0.39-1.60	0.51

Concurrent	OS	9	1350	0%	0.65	Fixed	0.55	0.45-0.67	<0.01
therapy	PFS	7	893	7%	0.38	Fixed	0.56	0.46-0.69	0.03

Supplementary Table 4. Publication bias analysis of literature reporting risk factors of OS and PFS in HCC patients accepted ICI treatment.

Risk factors	Outcome	Begg's test	Egger's test
Sex	OS	0.79	0.60
	PFS	0.68	0.83
AFP	OS	0.50	0.25
	PFS	0.66	0.45
Age	OS	0.05	0.01
	PFS	0.11	0.15
ALBI	OS	0.22	0.30
	PFS	0.32	0.18
BCLC stage	OS	0.01	0.01
	PFS	0.58	0.75
Child-Pugh stage	OS	1.00	0.75
	PFS	0.65	0.54
ECOG performance status	OS	0.18	0.04
	PFS	0.80	0.75
Viral infection	OS	0.52	0.61
	PFS	0.43	0.41
Liver cirrhosis	OS	0.80	0.59
	PFS	0.83	0.39
Vascular invasion	OS	0.05	0.08
	PFS	0.03	0.04
Tumor size	OS	0.88	0.79
	PFS	0.35	0.27
Tumor number	OS	0.24	0.03
	PFS	0.09	0.08
Extrahepatic metastasis	OS	0.86	0.46
	PFS	0.60	0.12
NLR	OS	0.01	<0.01
	PFS	0.24	0.18
PLR	OS	NA	NA
	PFS	0.50	0.80
Previous treatment	OS	0.41	0.77
	PFS	0.96	0.46
IrAE	OS	0.17	0.35
	PFS	0.60	0.69
Concurrent therapy	OS	1.00	0.32
	PFS	0.29	0.37

Supplementary Table 5. Subgroup analysis according to region of risk factors of OS and PFS in HCC patients accepted ICI treatment.

Risk factor	Subgroup	No. of articles on OS	Pooled HR for OS	Heterogeneity (I^2 and P value)	P-value for subgroup differences	No. of articles on PFS	Pooled HR for PFS	Heterogeneity (I^2 and P value)	P-value for subgroup differences
AFP									
	Asian	24	1.54 (1.35-1.76)	I^2=39% P=0.03	0.60	24	1.38 (1.20-1.59)	I^2=55% P<0.01	0.33
	Non-Asian	5	1.46 (1.22-1.74)	I^2=0% P=0.82		3	1.22 (1.00-1.49)	I^2=51% P=0.13	
ALBI									
	Asian	11	2.17 (1.89-2.49)	I^2=0% P=0.46	0.36	13	1.40 (1.23-1.58)	I^2=22% P=0.22	1.00
	Non-Asian	4	2.62 (1.79-3.85)	I^2=0% P=0.79		3	1.40 (1.04-1.89)	I^2=35% P=0.22	
NLR									
	Asian	7	1.41 (1.18-1.69)	I^2=38% P=0.14	0.74	8	1.47 (1.21-1.80)	I^2=32% P=0.17	0.16
	Non-Asian	3	1.54 (0.94-2.54)	I^2=57% P=0.10		3	1.19 (0.95-1.49)	I^2=0% P=0.88	
ECOG performance status									
	Asian	13	2.68 (2.02-3.56)	I^2=50% P=0.02	<0.01	13	1.85 (1.46-2.34)	I^2=52% P=0.01	0.23
	Non-Asian	5	1.54 (1.24-1.91)	I^2=28% P=0.24		2	1.16 (0.56-2.38)	I^2=89% P<0.01	
Child-Pugh stage									
	Asian	15	1.83 (1.37-2.45)	I^2=47% P=0.02	0.22	14	1.43 (1.20-1.70)	I^2=0% P=0.45	0.50
	Non-Asian	5	2.42 (1.72-3.40)	I^2=52% P=0.08		2	1.29 (1.02-1.64)	I^2=0% P=0.42	
BCLC stage									
	Asian	18	1.51 (1.37-2.45)	I^2=0% P=0.64	0.20	19	1.27 (1.13-1.43)	I^2=0% P=0.45	0.62
	Non-Asian	8	1.28 (1.07-1.54)	I^2=28% P=0.21		4	1.21 (1.05-1.40)	I^2=0% P=0.91	
Tumor number									
	Asian	9	1.75 (1.17-2.63)	I^2=59% P=0.01	0.27	8	1.37 (1.08-1.73)	I^2=35% P=0.15	0.97
	Non-Asian	1	1.08 (0.51-2.30)	NA		1	1.39 (0.69-2.81)	NA	
Vascular invasion									
	Asian	17	1.64 (1.39-1.94)	I^2=25% P=0.17	0.22	15	1.38 (1.14-1.66)	I^2=51% P=0.01	0.70
	Non-Asian	5	1.33	I^2=40%		3	2.01	I^2=84%	

		(0.97-1.79)	P=0.16			(0.30-13.41)	P<0.01	
Concurrent therapy								
Asian	9	0.55 (0.45-0.67)	I^2=0% P=0.67	NA	NA	NA	NA	NA
Non-Asian	NA	NA	NA		NA	NA	NA	

Supplementary Table 6. Baseline characteristics of patients in 2 validation cohorts.

Characteristics	Overall	Cohort 1	Cohort 2
n	204	105	99
Age, year	65.5 ± 13.0	73.7 ± 9.4	56.7± 10.5
Sex, n (%)			
Male	166 (81.4%)	84 (80%)	82 (82.8%)
Female	38 (18.6%)	21 (20%)	17 (17.2%)
ICI name, n (%)			
Atezolizumab	145 (71.1%)	105 (100.0%)	40 (40.4%)
Camrelizumab	23 (11.3%)	0 (0.0%)	23 (23.2%)
Sintilimab	23 (11.3%)	0 (0.0%)	23 (23.2%)
Tislelizumab	7 (3.4%)	0 (0.0%)	7 (7.1%)
Toripalimab	4 (2.0%)	0 (0.0%)	4 (4.0%)
Nivolumab	1 (0.5%)	0 (0.0%)	1 (1.0%)
Penpulimab	1 (0.5%)	0 (0.0%)	1 (1.0%)
AFP, n (%)			
≤400 ng/ml	140 (68.6%)	79 (75.2%)	61 (61.6%)
>400 ng/ml	64 (31.4%)	26 (24.8%)	38 (38.4%)
ALBI, n (%)			
≤I	89 (43.6%)	31 (29.5%)	58 (58.6%)
>I	115 (56.4%)	74 (70.5%)	41 (41.4%)
NLR, n (%)			
≤3	123 (60.3%)	72 (68.6%)	51 (51.5%)
>3	81 (39.7%)	33 (31.4%)	48 (48.5%)
ECOG performance status, n (%)			
≤0	155 (76%)	99 (94.3%)	56 (56.6%)
>0	49 (24%)	6 (5.7%)	43 (43.4%)
Child-Pugh stage, n (%)			
≤A	164 (80.4%)	105 (100%)	59 (59.6%)
>A	40 (19.6%)	0 (0%)	40 (40.4%)
BCLC stage, n (%)			
≤B	89 (43.6%)	50 (47.6%)	39 (39.4%)
>B	115 (56.4%)	55 (52.4%)	60 (60.6%)
Tumor number, n (%)			
≤1 (≤5)	95 (46.6%)	64 (61.0%)	31 (31.3%)
>1 (>5)	109 (53.4%)	41 (39.0%)	68 (68.7%)
Vascular invasion, n (%)			
YES	43 (21.1%)	20 (19%)	23 (23.2%)
NO	161 (78.9%)	85 (81%)	76 (76.8%)
Concurrent therapy			
YES	197 (96.6%)	105 (100.0%)	92 (92.9%)
NO	7 (3.4%)	0 (0.0%)	7 (7.1%)
Follow-up, months			

	8.1 (5.0, 13.2)	6.4 (4.6, 9.5)	12.2 (6.25, 21.6)

Data are mean ± SD, median (IQR), or n (%).

Abbreviations in Supplementary Tables

P, prospective study; R, retrospective study; PD-1, programmed cell death-1; PD-L1, programmed death ligand-1; Mixed, refers to the inclusion of two or three ICI types; ICI, immune checkpoint inhibitors; OS, overall survival; PFS, progression-free survival; NOS, Newcastle - Ottawa Scale; AFP, alpha-fetoprotein; ALBI, albumin-bilirubin score; ECOG, Eastern Cooperative Oncology Group; IrAE, immune-related adverse events; NLR, neutrophil-to-lymphocyte ratio; PLR, platelet-to-lymphocyte ratio; NA, not available.

3. Supplementary Figure

Supplementary Figure 1. The results of meta and sensitivity analysis of AFP on OS and PFS.

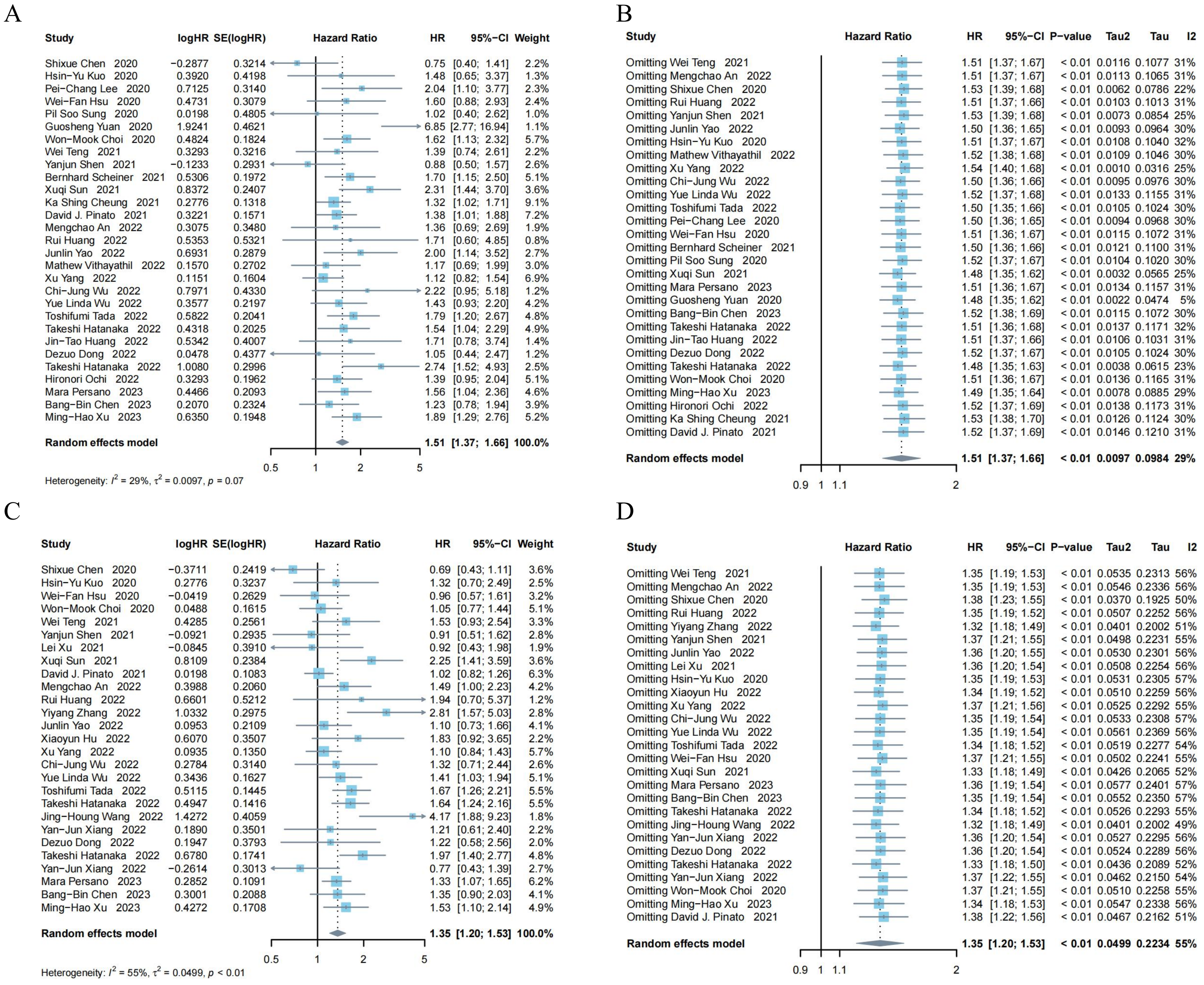

Supplementary Figure 1 **A**. The pooled results of the correlation of AFP with OS. **B**. The sensitivity analysis plot of AFP for OS. **C**. The pooled results of the correlation of AFP with PFS. **D**. The sensitivity analysis plot of AFP for PFS.

Supplementary Figure 2. The results of meta and sensitivity analysis of ALBI on OS and PFS.

A

B

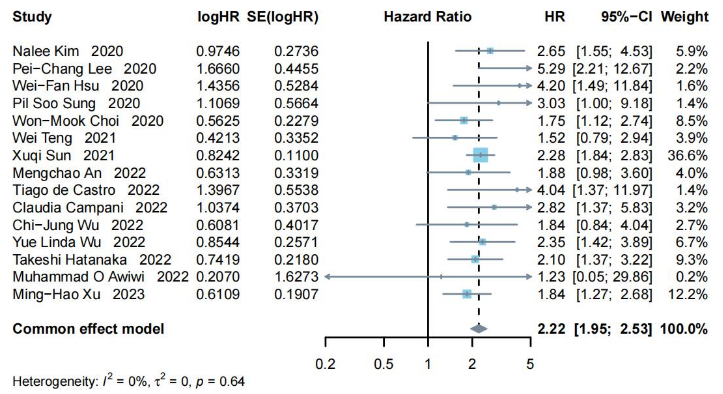

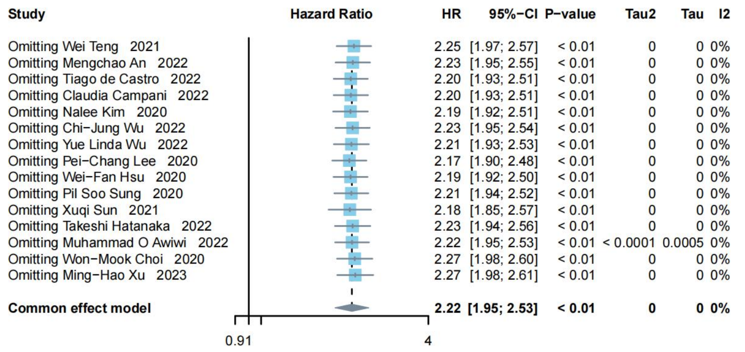

C

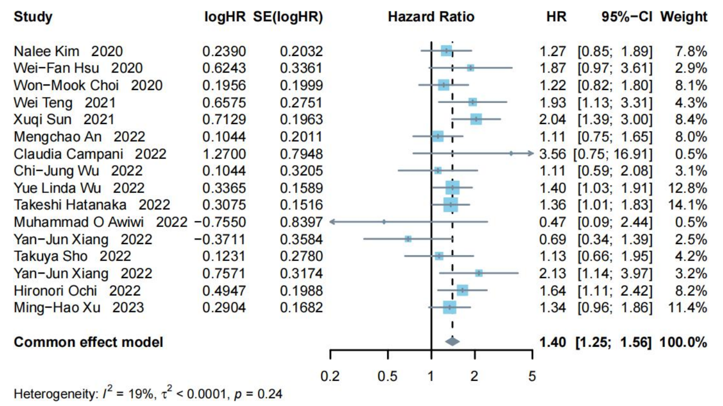

D

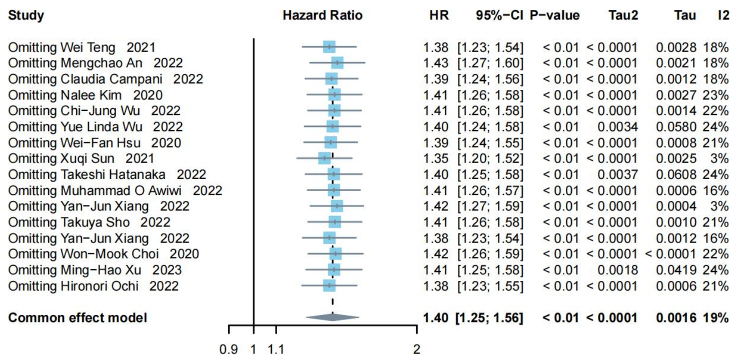

Supplementary Figure 2 **A**. The pooled results of the correlation of ALBI with OS. **B**. The sensitivity analysis plot of ALBI for OS. **C**. The pooled results of the correlation of ALBI with PFS. **D**. The sensitivity analysis plot of ALBI for PFS.

Supplementary Figure 3. The results of meta and sensitivity analysis of NLR on OS and PFS.

A

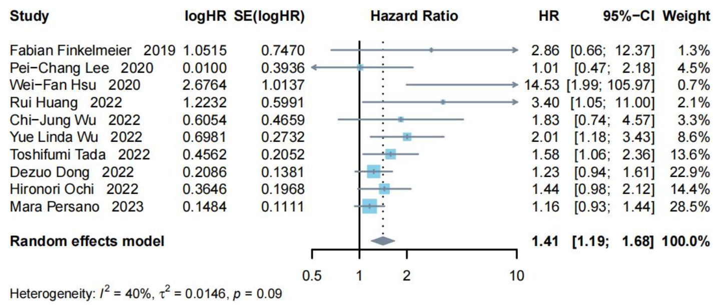

B

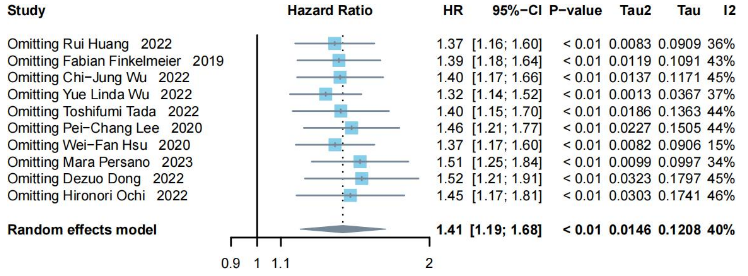

C

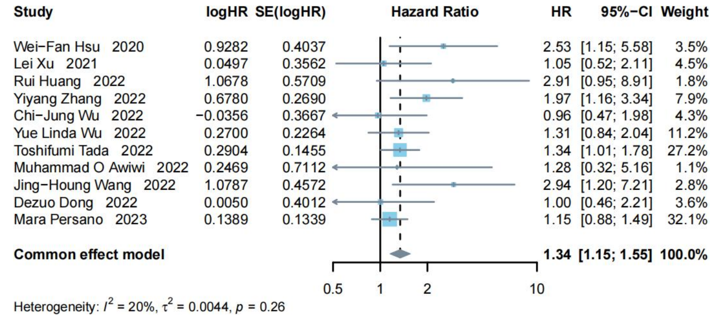

D

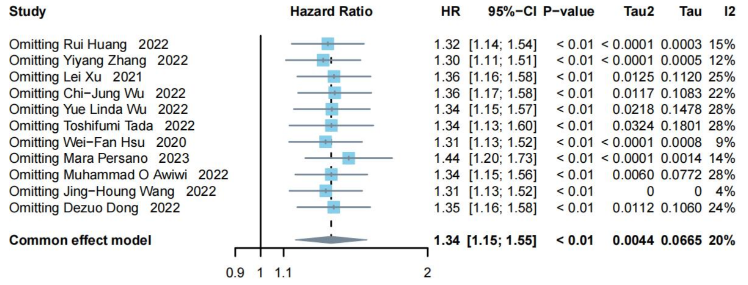

Supplementary Figure 3 **A**. The pooled results of the correlation of NLR with OS. **B**. The sensitivity analysis plot of NLR for OS. **C**. The pooled results of the correlation of NLR with PFS. **D**. The sensitivity analysis plot of NLR for PFS.

Supplementary Figure 4. The results of meta and sensitivity analysis of ECOG performance status on OS and PFS.

A

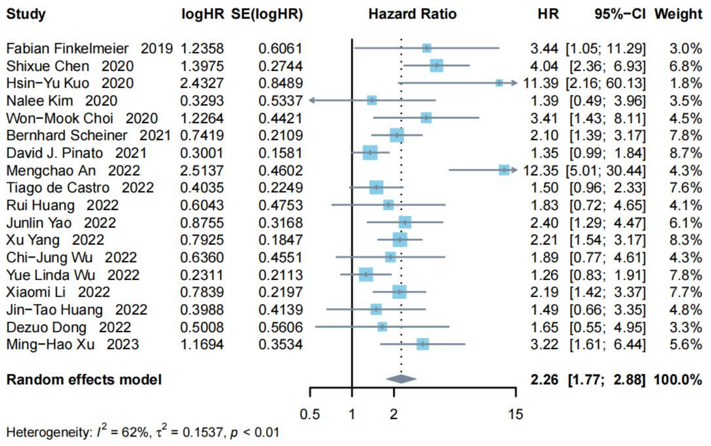

B

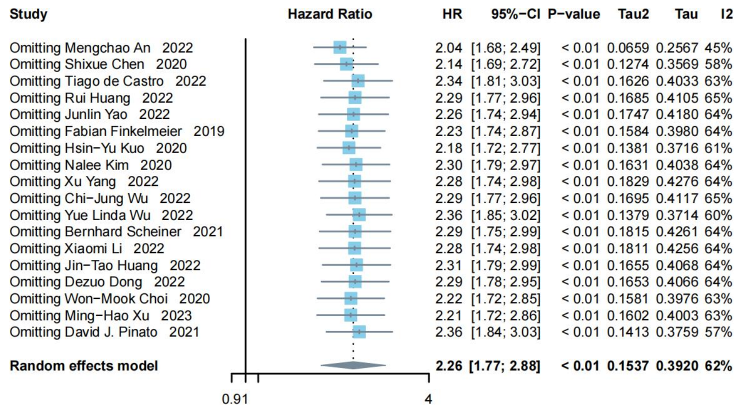

C

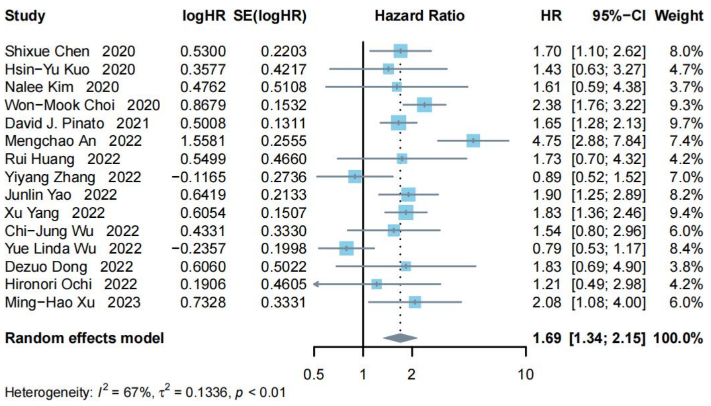

D

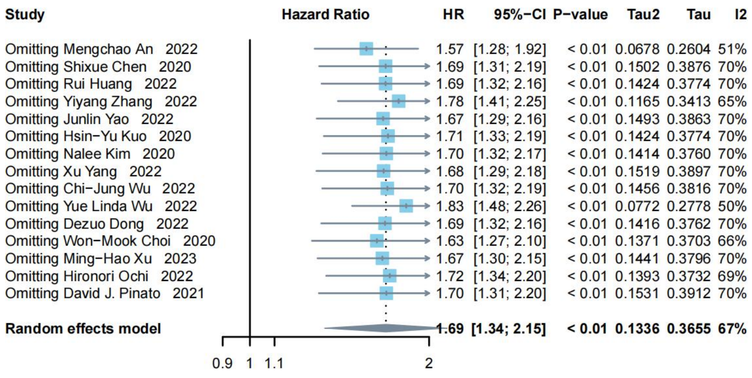

Supplementary Figure 4 **A**. The pooled results of the correlation of ECOG performance status with OS. **B**. The sensitivity analysis plot of ECOG performance status for OS. **C**. The pooled results of the correlation of ECOG performance status with PFS. **D**. The sensitivity analysis plot of ECOG performance status for PFS.

Supplementary Figure 5. The results of meta and sensitivity analysis of Child-Pugh stage on OS and PFS.

A

B

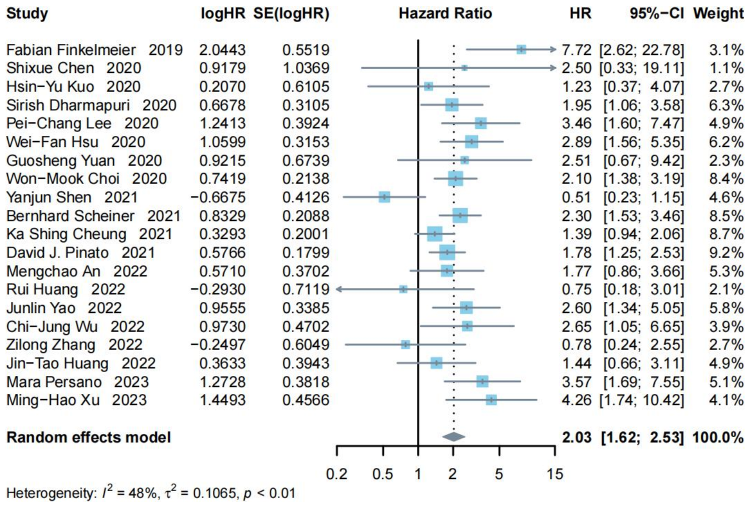

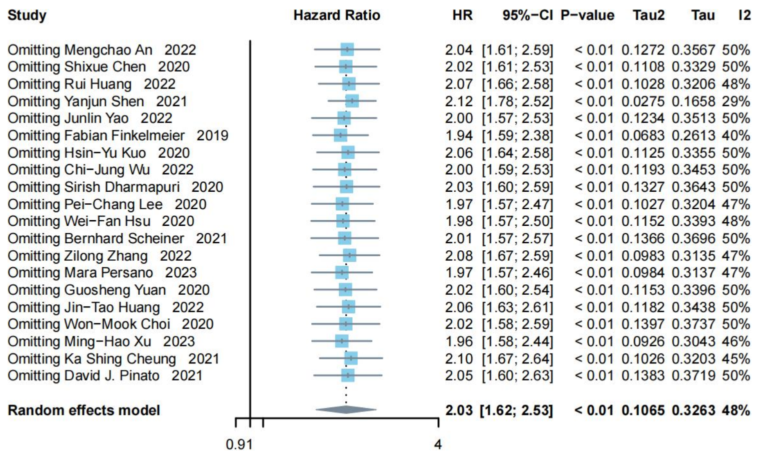

C

D

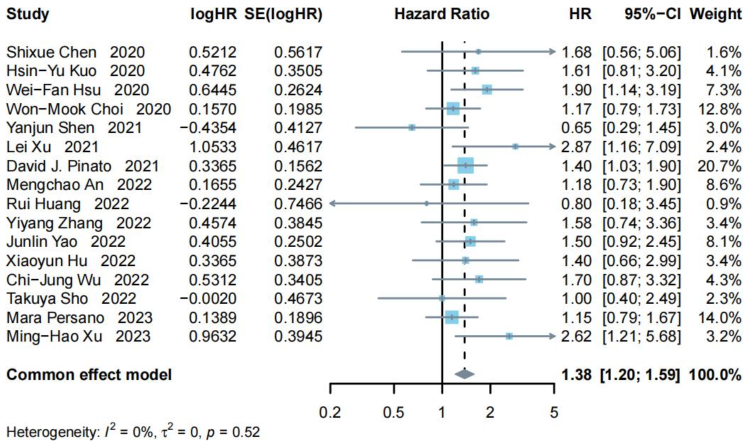

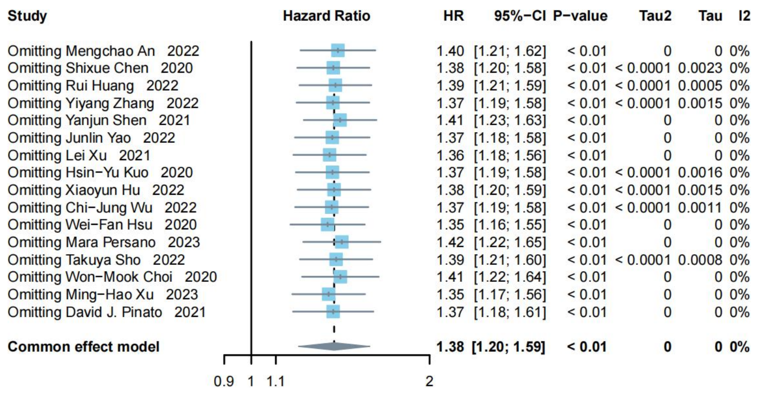

Supplementary Figure 5 **A**. The pooled results of the correlation of Child-Pugh stage with OS. **B**. The sensitivity analysis plot of Child-Pugh stage for OS. **C**. The pooled results of the correlation of Child-Pugh stage with PFS. **D**. The sensitivity analysis plot of Child-Pugh stage for PFS.

Supplementary Figure 6. The results of meta and sensitivity analysis of BCLC stage on OS and PFS.

A

B

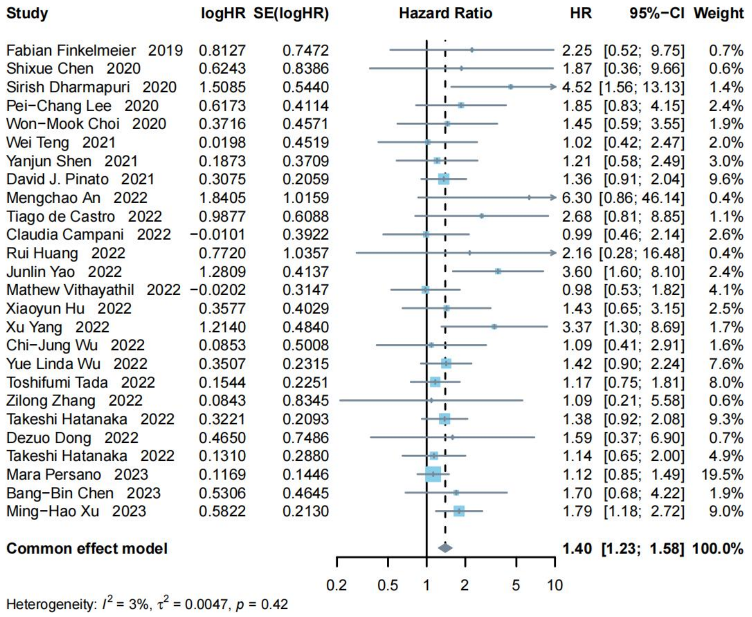

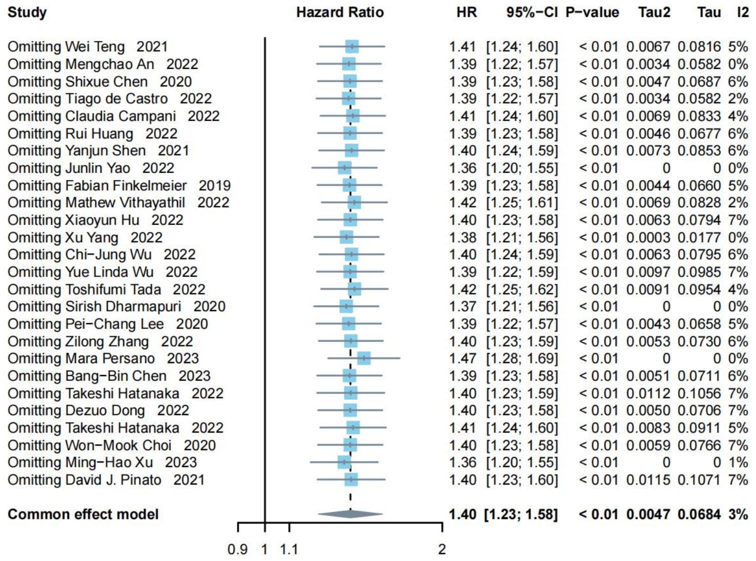

C

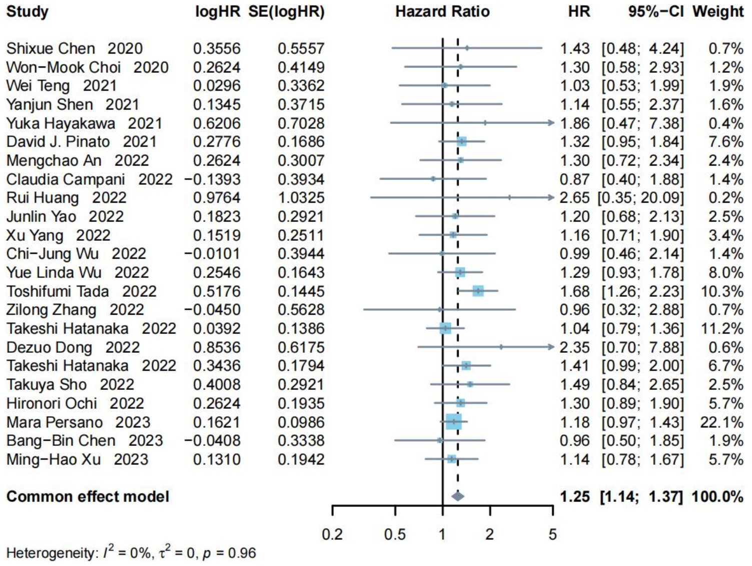

D

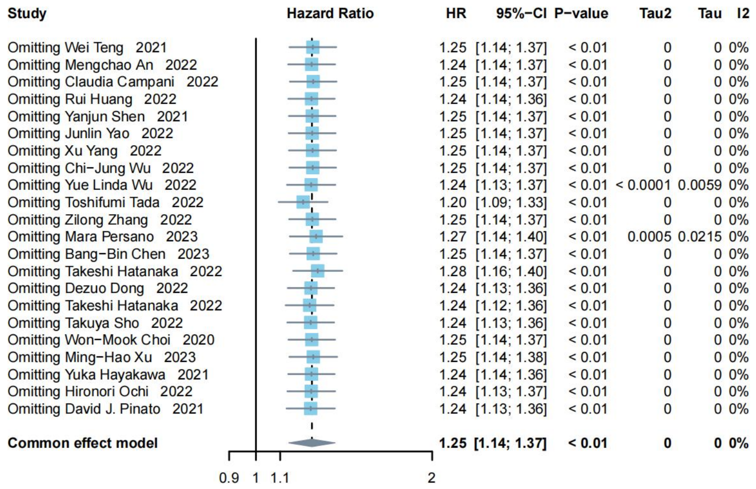

Supplementary Figure 4 **A**. The pooled results of the correlation of BCLC stage with OS. **B**. The sensitivity analysis plot of BCLC stage for OS. **C**. The pooled results of the correlation of BCLC stage with PFS. **D**. The sensitivity analysis plot of BCLC stage for PFS.

Supplementary Figure 7. The results of meta and sensitivity analysis of tumor number on OS and PFS.

A

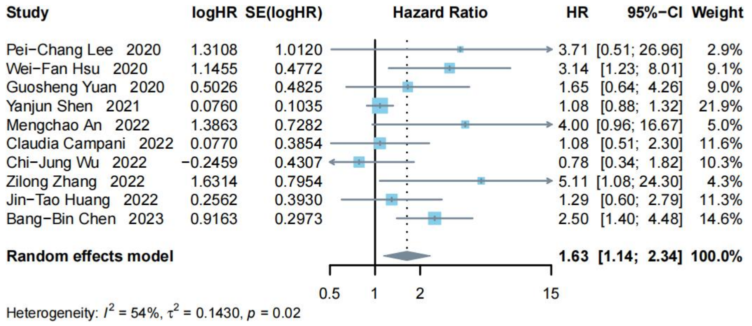

B

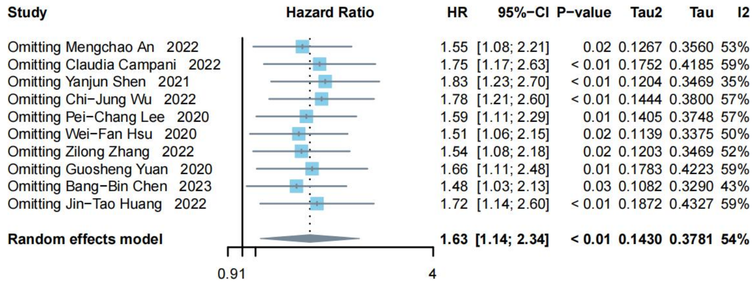

C

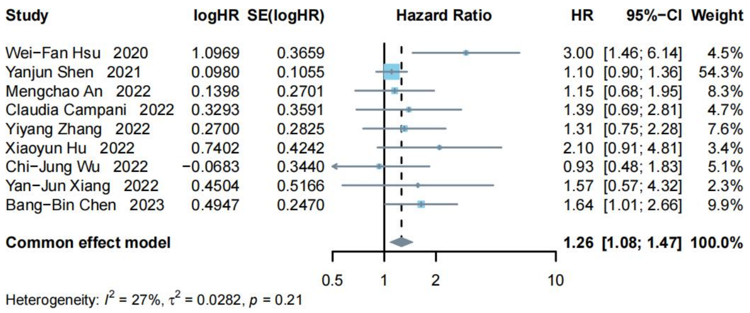

D

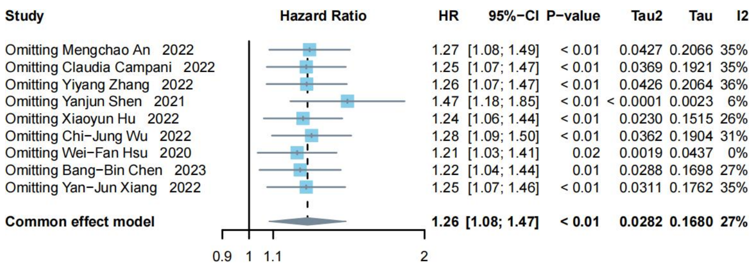

Supplementary Figure 7 **A**. The pooled results of the correlation of tumor number with OS. **B**. The sensitivity analysis plot of tumor number for OS. **C**. The pooled results of the correlation of tumor number with PFS. **D**. The sensitivity analysis plot of tumor number for PFS.

Supplementary Figure 8. The results of meta and sensitivity analysis of vascular invasion on OS and PFS.

A

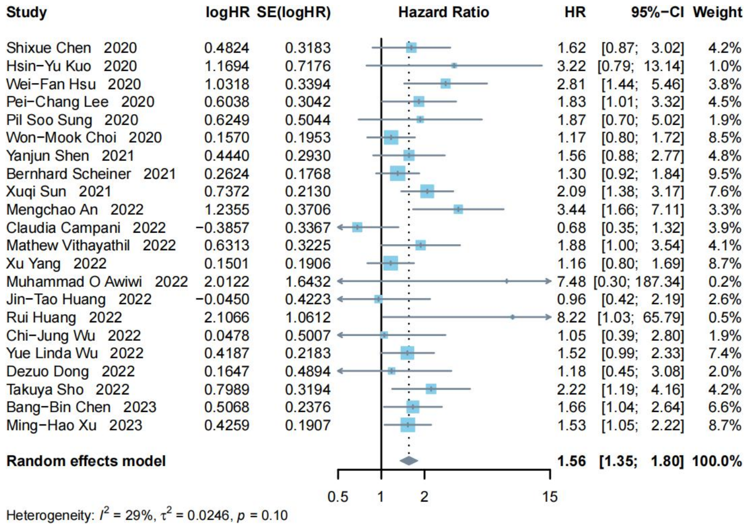

B

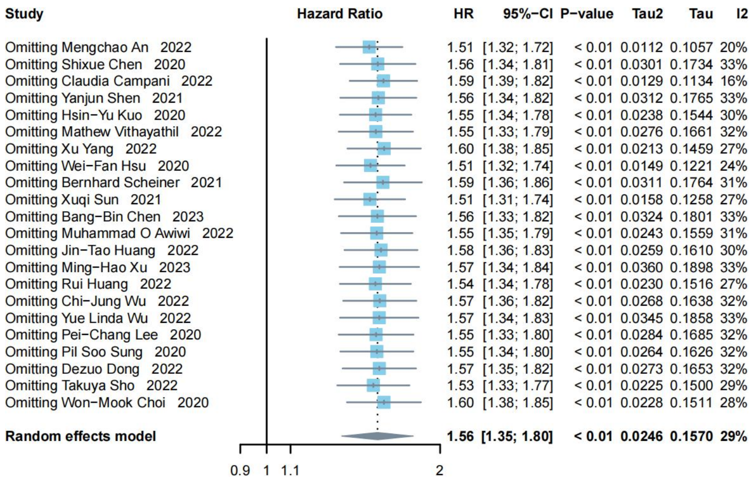

C

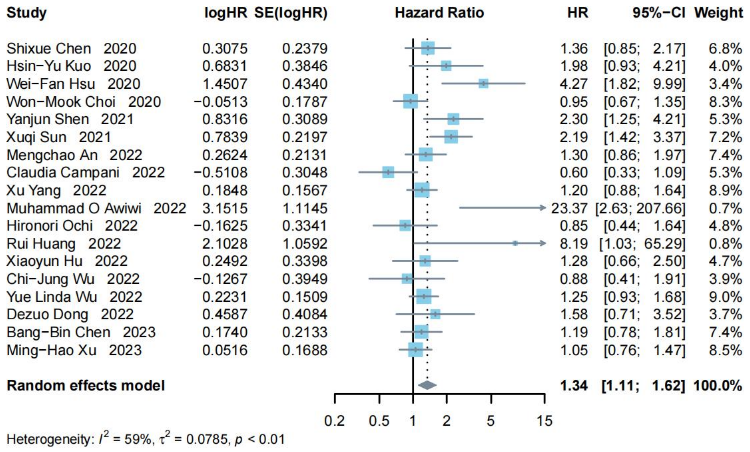

D

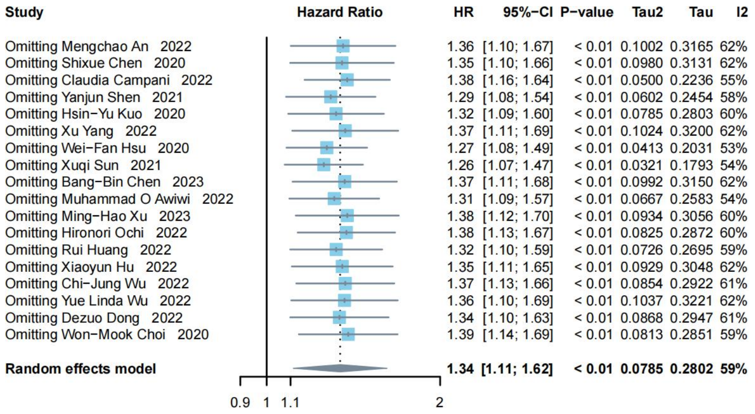

Supplementary Figure 8 **A**. The pooled results of the correlation of vascular invasion with OS. **B**. The sensitivity analysis plot of vascular invasion for OS. **C**. The pooled results of the correlation of vascular invasion with PFS. **D**. The sensitivity analysis plot of vascular invasion for PFS.

Supplementary Figure 9. The results of meta and sensitivity analysis of concurrent therapy on OS and PFS.

A

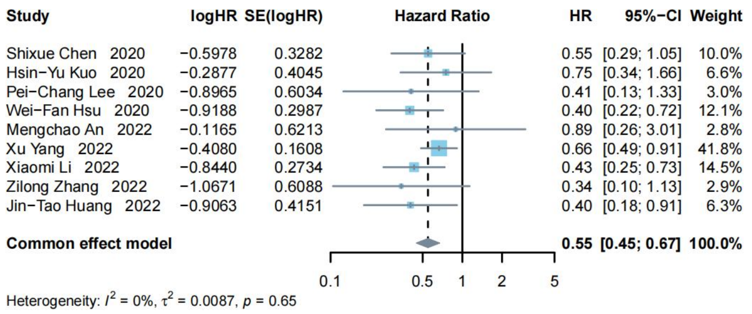

B

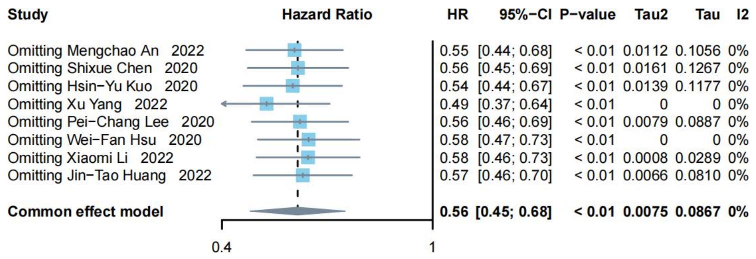

C

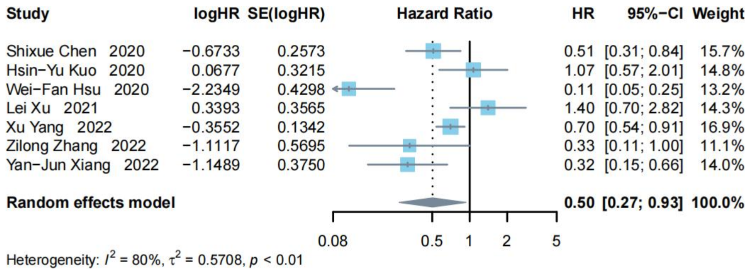

D

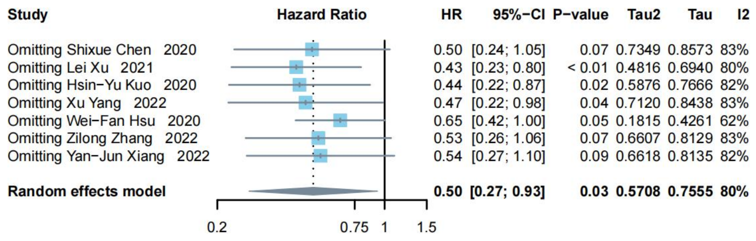

Supplementary Figure 9 **A**. The pooled results of the correlation of concurrent therapy with OS. **B**. The sensitivity analysis plot of concurrent therapy for OS. **C**. The pooled results of the correlation of concurrent therapy with PFS. **D**. The sensitivity analysis plot of concurrent therapy for PFS.

Supplementary Figure 10

A

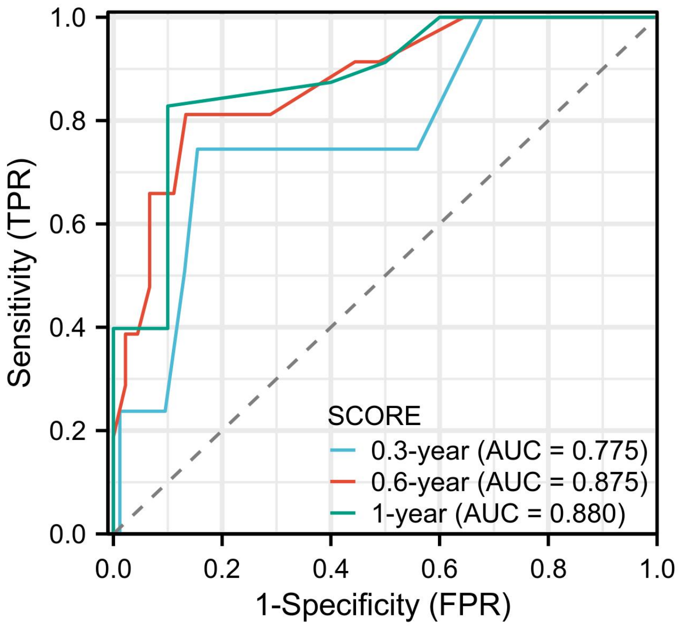

B

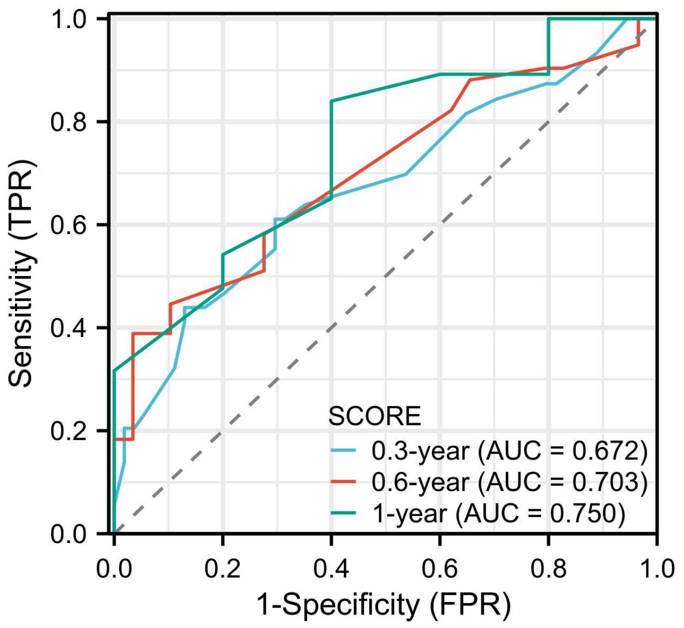

C

D

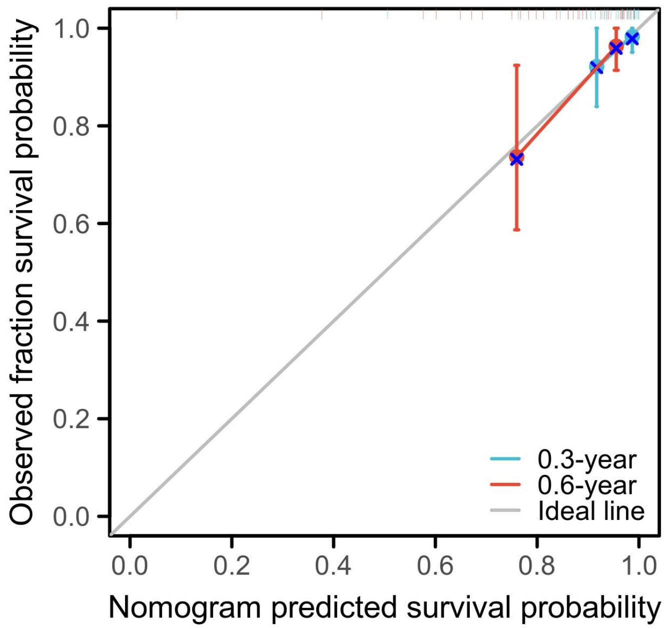

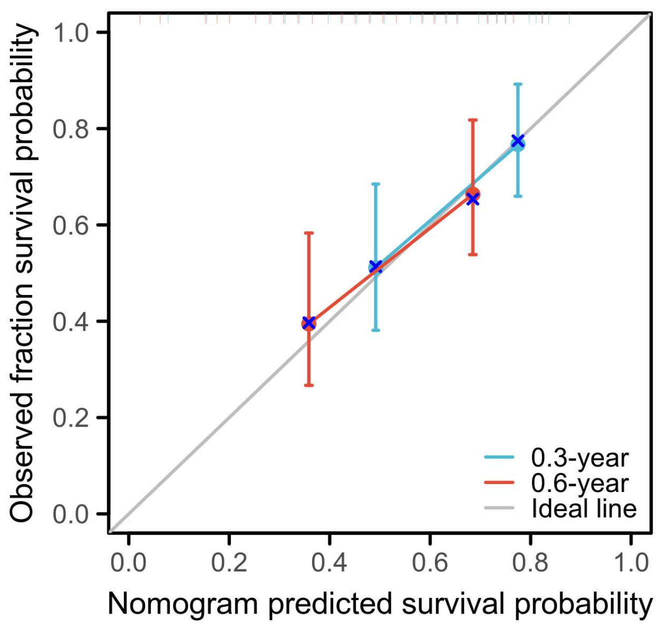

E

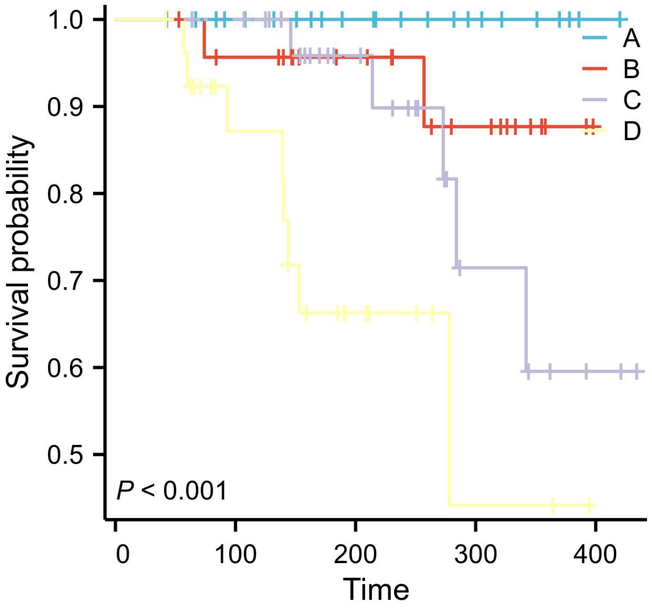

F

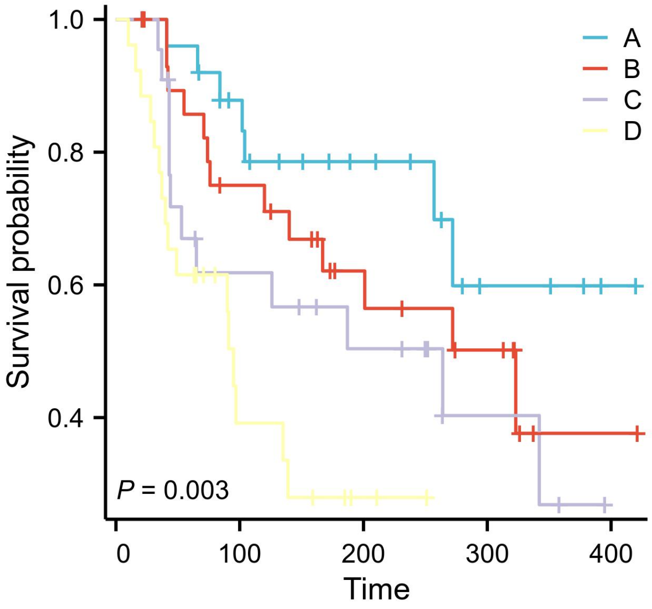

Supplementary Figure 10 **A**. The ROC curves for OS prediction model in validation cohort 1. **B.** The ROC curves for PFS prediction model in validation cohort 1. **C**. The calibration curves for the OS prediction model in validation cohort 1. **D**. The calibration curves for the PFS prediction model in validation cohort 1. **E**. The KM curves of OS for the four risk groups in validation cohort 1. **F**.The KM curves of PFS for the four risk groups in validation cohort 1.

Supplementary Figure 11

A

B

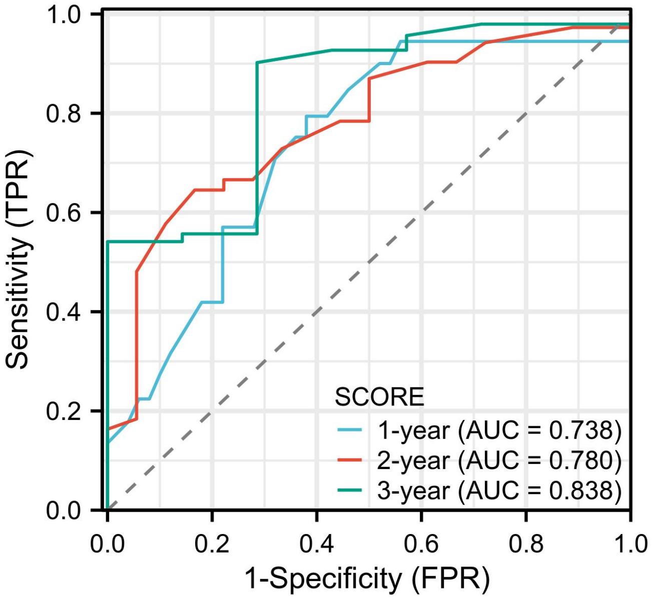
Sensitivity (TPR)
1-Specificity (FPR)
SCORE
1-year (AUC = 0.738)
2-year (AUC = 0.780)
3-year (AUC = 0.838)

C

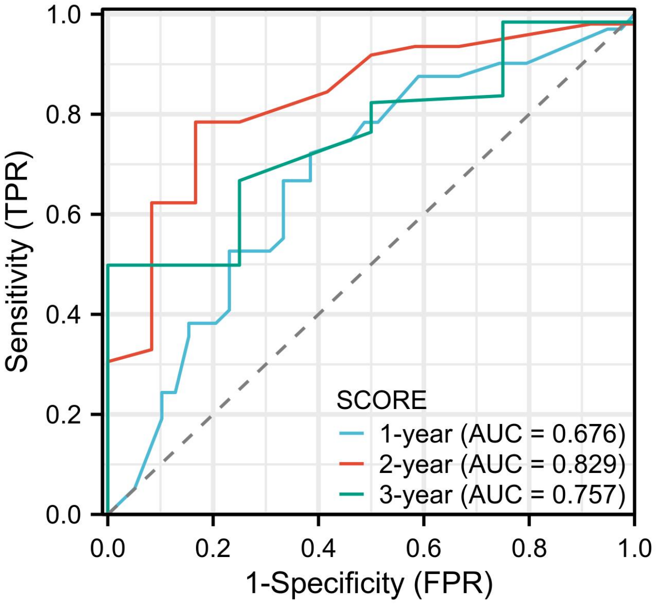
Sensitivity (TPR)
1-Specificity (FPR)
SCORE
1-year (AUC = 0.676)
2-year (AUC = 0.829)
3-year (AUC = 0.757)

D

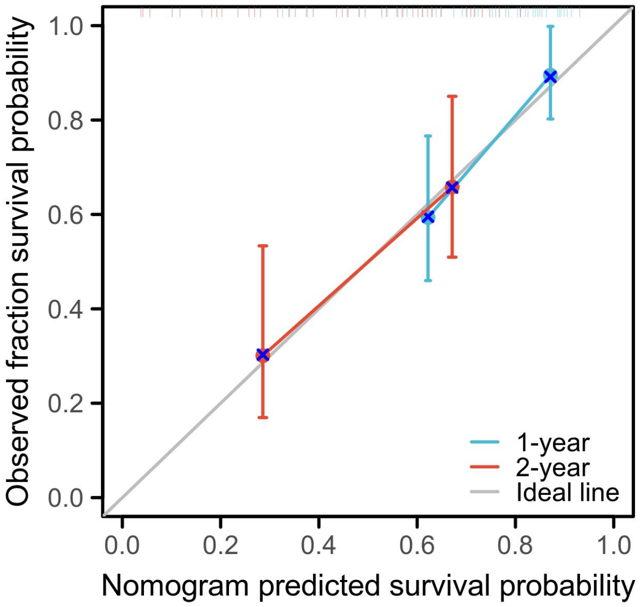
Observed fraction survival probability
Nomogram predicted survival probability
1-year
2-year
Ideal line

E

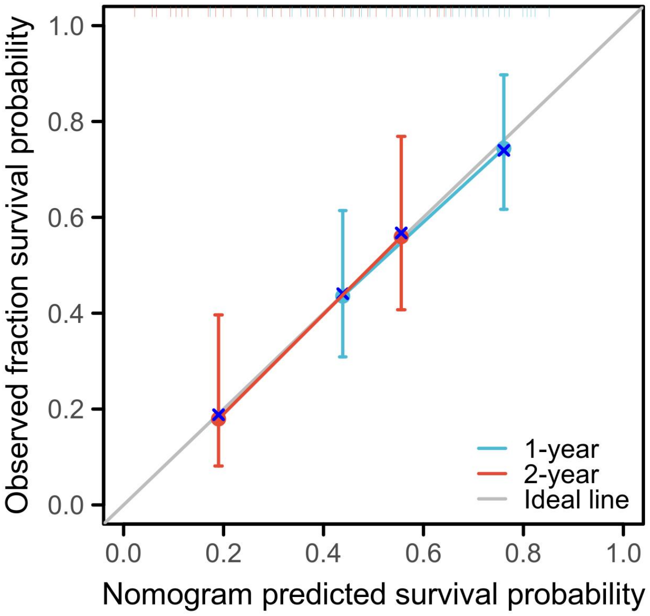
Observed fraction survival probability
Nomogram predicted survival probability
1-year
2-year
Ideal line

F

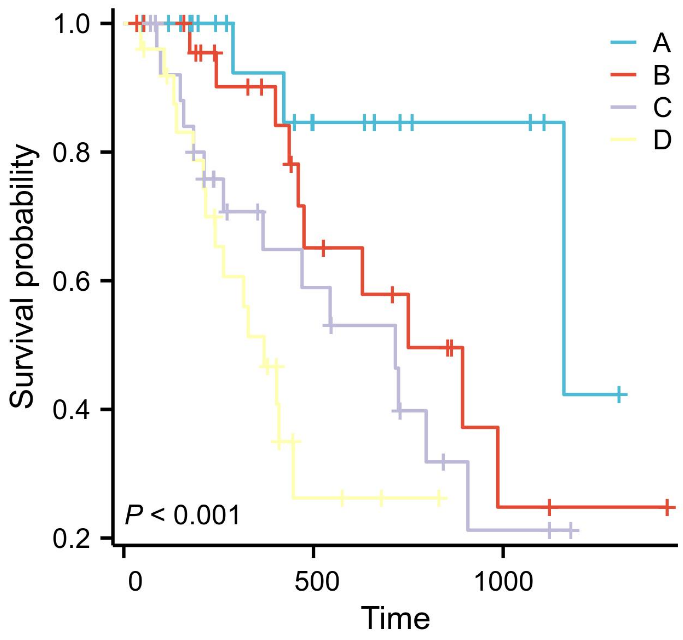

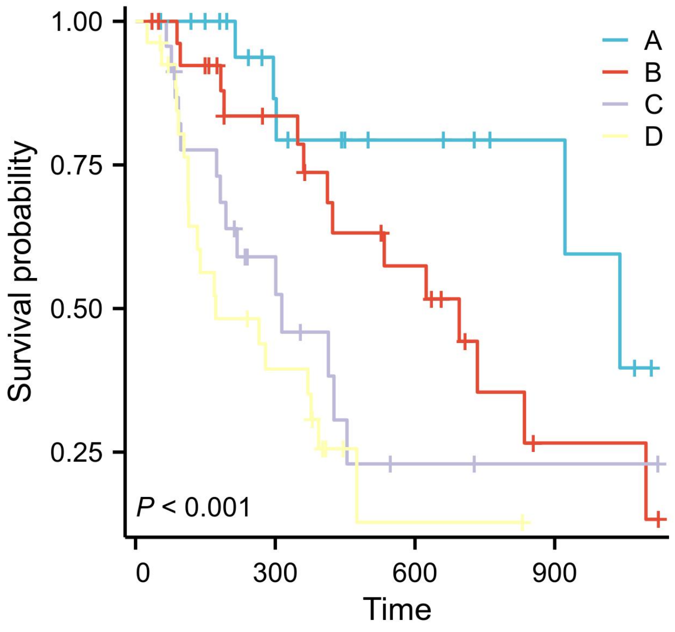

Supplementary Figure 11 **A**. The ROC curves for OS prediction model in validation cohort 2. **B.** The ROC curves for PFS prediction model in validation cohort 2. **C**. The calibration curves for the OS prediction model in validation cohort 2. **D**. The calibration curves for the PFS prediction model in validation cohort 2. **E**. The KM curves of OS for the four risk groups in validation cohort 2. **F**.The KM curves of PFS for the four risk groups in validation cohort 2.

References

1. Teng W, Lin CC, Ho MM, Lui KW, Wang SF, Hsu CW, Lin SM: **Alpha-fetoprotein response at different time-points is associated with efficacy of nivolumab monotherapy for unresectable hepatocellular carcinoma**. *AM J CANCER RES* 2021, **11**(5):2319-2330.
2. An M, Wang W, Zhang J, Till BG, Zhao L, Huang H, Yang Y, Li T, Han L, Zhang X *et al*: **Association of hepatitis B virus DNA levels with overall survival for advanced hepatitis B virus-related hepatocellular carcinoma under immune checkpoint inhibitor therapy**. *Cancer Immunology, Immunotherapy* 2023, **72**(2):385-395.
3. Chen S, Huang Z, Jia W, Tao H, Zhang S, Ma J, Liu Z, Wang J, Wang L, Cui P *et al*: **Association of the pretreatment lung immune prognostic index with survival outcomes in advanced hepatocellular carcinoma patients treated with pd-1 inhibitors**. *Journal of Hepatocellular Carcinoma* 2020, **7**:289-299.
4. de Castro T, Jochheim LS, Bathon M, Welland S, Scheiner B, Shmanko K, Roessler D, Khaled NB, Jeschke M, Ludwig JM *et al*: **Atezolizumab and bevacizumab in patients with advanced hepatocellular carcinoma with impaired liver function and prior systemic therapy: a real-world experience**. *THER ADV MED ONCOL* 2022, **14**.
5. Campani C, Bamba-Funck J, Campion B, Sidali S, Blaise L, Ganne-Carrie N, Demory A, Sutter O, Larrey E, Evain M *et al*: **Baseline ALBI score and early variation of serum AFP predicts outcomes in patients with HCC treated by atezolizumab-bevacizumab**. *LIVER INT* 2023, **43**(3):708-717.
6. Huang R, Zheng Y, Zou W, Liu C, Liu J, Yue J: **Blood Biomarkers Predict Survival Outcomes in Patients with Hepatitis B Virus-Induced Hepatocellular Carcinoma Treated with PD-1 Inhibitors**. *J IMMUNOL RES* 2022, **2022**:3781109.
7. Zhang Y, Lu L, He Z, Xu Z, Xiang Z, Nie RC, Lin W, Chen W, Zhou J, Yin Y *et al*: **C-Reactive Protein Levels Predict Responses to PD-1 Inhibitors in Hepatocellular Carcinoma Patients**. *FRONT IMMUNOL* 2022, **13**:808101.
8. Shen Y, Wang H, Wei J, Li W: **Early prediction of objective response of fibrinogen in a real-world cohort of hepatocellular carcinoma cases treated by programmed cell death receptor-1 and lenvatinib**. *ONCOTARGETS THER* 2021, **14**:5019-5026.
9. Yao J, Zhu X, Wu Z, Wei Q, Cai Y, Zheng Y, Hu X, Hu H, Zhang X, Pan H *et al*: **Efficacy and safety of PD-1 inhibitor combined with antiangiogenic therapy for unresectable hepatocellular carcinoma: A multicenter retrospective study**. *CANCER MED-US* 2022, **11**(19):3612-3622.
10. Finkelmeier F, Czauderna C, Perkhofer L, Ettrich TJ, Trojan J, Weinmann A, Marquardt JU, Vermehren J, Waidmann O: **Feasibility and safety of nivolumab in advanced hepatocellular carcinoma: real-life experience from three German centers**. *J Cancer Res Clin Oncol* 2019, **145**(1):253-259.
11. Xu L, Leng C, Chen L, Dong H, Chen Y, Chen X: **Hypothyroidism is a predictive factor of superior antitumour efficacy of programmed death 1 inhibitors in hepatocellular carcinoma**. *INT J CANCER* 2022, **150**(3):472-481.
12. Kuo HY, Chiang NJ, Chuang CH, Chen CY, Wu IC, Chang TT, Tsai HM, Lin YJ: **Impact of Immune Checkpoint Inhibitors with or without a Combination of Tyrosine Kinase Inhibitors on Organ-Specific Efficacy and Macrovascular Invasion in Advanced Hepatocellular Carcinoma**. *ONCOL RES TREAT* 2020, **43**(5):211-220.
13. Ng KYY, Tan SH, Tan JJE, Tay DSH, Lee AWX, Ang AJS, Wong LWJ, Choo SP, Tai DWM, Lee JJX: **Impact of Immune-Related Adverse Events on Efficacy of Immune Checkpoint Inhibitors in Patients with Advanced Hepatocellular Carcinoma**. *Liver Cancer* 2022, **11**(1):9-21.
14. Vithayathil M, D'Alessio A, Fulgenzi CAM, Nishida N, Schönlein M, von Felden J, Schulze K, Wege H, Saeed A, Wietharn B *et al*: **Impact of older age in patients receiving atezolizumab and bevacizumab for hepatocellular carcinoma**. *LIVER INT* 2022, **42**(11):2538-2547.
15. Kim N, Yu JI, Park HC, Yoo GS, Choi C, Hong JY, Lim HY, Lee J, Choi MS, Lee JE *et al*: **Incorporating sarcopenia and inflammation with radiation therapy in patients with hepatocellular carcinoma treated with nivolumab**. *Cancer Immunol*

Immunother 2021, **70**(6):1593-1603.
16. Hu X, Li R, Li Q, Zang M, Yuan G, Chen J: **Interaction between baseline HBV loads and the prognosis of patients with HCC receiving anti-PD-1 in combination with antiangiogenic therapy undergoing concurrent TAF prophylaxis**. *BMC INFECT DIS* 2022, **22**(1):614.
17. Yang X, Chen B, Wang Y, Wang Y, Long J, Zhang N, Xue J, Xun Z, Zhang L, Cheng J *et al*: **Lenvatinib plus PD-1 inhibitors in 378 unresectable hepatocellular carcinoma: A large real-world study from two centers**. *J CLIN ONCOL* 2022, **40**(16).
18. Wu CJ, Lee PC, Hung YW, Lee CJ, Chi CT, Lee IC, Hou MC, Huang YH: **Lenvatinib plus pembrolizumab for systemic therapy-naïve and -experienced unresectable hepatocellular carcinoma**. *Cancer Immunology, Immunotherapy* 2022, **71**(11):2631-2643.
19. Wu YL, Fulgenzi CAM, D Alessio A, Cheon J, Nishida N, Saeed A, Wietharn B, Cammarota A, Pressiani T, Personeni N *et al*: **Neutrophil-to-Lymphocyte and Platelet-to-Lymphocyte Ratios as Prognostic Biomarkers in Unresectable Hepatocellular Carcinoma Treated with Atezolizumab plus Bevacizumab**. *CANCERS* 2022, **14**(23).
20. Tada T, Kumada T, Hiraoka A, Kariyama K, Tani J, Hirooka M, Takaguchi K, Atsukawa M, Fukunishi S, Itobayashi E *et al*: **New prognostic system based on inflammation and liver function predicts prognosis in patients with advanced unresectable hepatocellular carcinoma treated with atezolizumab plus bevacizumab: A validation study**. *CANCER MED-US*.
21. Dharmapuri S, Ozbek U, Lin J, Sung M, Schwartz M, Branch AD, Ang C: **Predictive value of neutrophil to lymphocyte ratio and platelet to lymphocyte ratio in advanced hepatocellular carcinoma patients treated with anti-PD-1 therapy**. *CANCER MED-US* 2020, **9**(14):4962-4970.
22. Lee PC, Chao Y, Chen MH, Lan KH, Lee CJ, Lee IC, Chen SC, Hou MC, Huang YH: **Predictors of response and survival in immune checkpoint inhibitor-treated unresectable hepatocellular carcinoma**. *CANCERS* 2020, **12**(1).
23. Hsu WF, Chuang PH, Chen CK, Wang HW, Tsai MH, Su WP, Chen HY, Yang CY, Lin CC, Huang GT *et al*: **Predictors of response and survival in patients with unresectable hepatocellular carcinoma treated with nivolumab: Real-world experience**. *AM J CANCER RES* 2020, **10**(12):4547-4560.
24. Scheiner B, Pomej K, Kirstein MM, Hucke F, Finkelmeier F, Waidmann O, Himmelsbach V, Schulze K, von Felden J, Fründt TW *et al*: **Prognosis of patients with hepatocellular carcinoma treated with immunotherapy - development and validation of the CRAFITY score**. *J HEPATOL* 2022, **76**(2):353-363.
25. Li X, Sun W, Ding X, Li W, Chen J: **Prognostic model of immune checkpoint inhibitors combined with anti-angiogenic agents in unresectable hepatocellular carcinoma**. *FRONT IMMUNOL* 2022, **13**:1060051.
26. Zhang Z, Liang Y, Zhong D, Dai Z, Shang J, Lai C, Zou H, Yao Y, Feng T, Huang X: **Prognostic value of inflammation-immunity-nutrition score in patients with hepatocellular carcinoma treated with anti-PD-1 therapy**. *J CLIN LAB ANAL* 2022, **36**(5):e24336.
27. Sung PS, Jang JW, Lee J, Lee SK, Lee HL, Yang H, Nam HC, Lee SW, Bae SH, Choi JY *et al*: **Real-World Outcomes of Nivolumab in Patients With Unresectable Hepatocellular Carcinoma in an Endemic Area of Hepatitis B Virus Infection**. *FRONT ONCOL* 2020, **10**.
28. Sun X, Mei J, Lin W, Yang Z, Peng W, Chen J, Zhang Y, Xu L, Chen M: **Reductions in AFP and PIVKA-II can predict the efficiency of anti-PD-1 immunotherapy in HCC patients**. *BMC CANCER* 2021, **21**(1).
29. Persano M, Rimini M, Cascinu S, Scartozzi M, Casadei Gardini A: **Role of the prognostic nutritional index in predicting survival in advanced hepatocellular carcinoma treated with atezolizumab plus bevacizumab**. *Immuno-Oncology and Technology* 2022, **16**.
30. Yuan G, Cheng X, Li Q, Zang M, Huang W, Fan W, Wu T, Ruan J, Dai W, Yu W *et al*: **Safety and efficacy of camrelizumab combined with apatinib for advanced hepatocellular carcinoma with portal vein tumor thrombus: A multicenter retrospective study**. *ONCOTARGETS THER* 2020, **13**:12683-12693.
31. Chen BB, Liang PC, Shih TTF, Liu TH, Shen YC, Lu LC, Lin ZZ, Hsu C, Hsu CH, Cheng AL *et al*: **Sarcopenia and myosteatosis are associated with survival in patients receiving immunotherapy for advanced hepatocellular carcinoma**. *EUR RADIOL* 2023, **33**(1):512-522.

32. Hatanaka T, Naganuma A, Hiraoka A, Tada T, Hirooka M, Kariyama K, Tani J, Atsukawa M, Takaguchi K, Itobayashi E *et al*: **The hepatocellular carcinoma modified Gustave Roussy Immune score (HCC-GRIm score) as a novel prognostic score for patients treated with atezolizumab and bevacizumab: A multicenter retrospective analysis**. *Cancer Med* 2023, **12**(4):4259-4269.
33. Awiwi MO, Elsayes KM, Mohamed YI, Altameemi L, Gjoni M, Irshad OM, Ahmed AS, Kaseb AO, Salem U: **The Prognostic Value of Baseline Clinical and Radiologic Imaging Features in Patients with Unresectable Hepatocellular Carcinoma Treated with Atezolizumab Plus Bevacizumab**. *Journal of Hepatocellular Carcinoma* 2022, **9**:913-927.
34. Wang JH, Chen YY, Kee KM, Wang CC, Tsai MC, Kuo YH, Hung CH, Li WF, Lai HL, Chen YH: **The Prognostic Value of Neutrophil-to-Lymphocyte Ratio and Platelet-to-Lymphocyte Ratio in Patients with Hepatocellular Carcinoma Receiving Atezolizumab Plus Bevacizumab**. *CANCERS* 2022, **14**(2).
35. Huang J, Zhong B, Jiang N, Li W, Zhang S, Yin Y, Yang J, Shen J, Wang W, Zhu X: **Transarterial Chemoembolization Combined with Immune Checkpoint Inhibitors Plus Tyrosine Kinase Inhibitors versus Immune Checkpoint Inhibitors Plus Tyrosine Kinase Inhibitors for Advanced Hepatocellular Carcinoma**. *JOURNAL OF HEPATOCELLULAR CARCINOMA* 2022, **9**:1217-1228.
36. Pinato DJ, Wang Y, Marron TU, Saeed A, Jun T, Naqash AR, Dharmapuri S, Khan U, Benevento F, Navaid M *et al*: **Treatment-related toxicity predicts for improved outcome in patients with hepatocellular carcinoma (HCC) treated with immune checkpoint inhibitor therapy**. *J HEPATOL* 2020, **73**:S40-S41.
37. Cheung KS, Lam LK, Seto WK, Leung WK: **Use of Antibiotics during Immune Checkpoint Inhibitor Treatment Is Associated with Lower Survival in Hepatocellular Carcinoma**. *LIVER CANCER* 2021, **10**(6):606-614.
38. Ochi H, Kurosaki M, Joko K, Mashiba T, Tamaki N, Tsuchiya K, Marusawa H, Tada T, Nakamura S, Narita R *et al*: **Usefulness of neutrophil-to-lymphocyte ratio in predicting progression and survival outcomes after atezolizumab‐bevacizumab treatment for hepatocellular carcinoma**. *HEPATOL RES* 2023, **53**(1):61-71.
39. Lewis S, Cedillo MA, Lee KM, Bane O, Hectors S, Ma W, Wang P, Stocker D, Morris DV, Pinato D *et al*: **Comparative assessment of standard and immune response criteria for evaluation of response to PD-1 monotherapy in unresectable HCC**. *Abdom Radiol (NY)* 2022, **47**(3):969-980.
40. Hayakawa Y, Tsuchiya K, Kurosaki M, Yasui Y, Kaneko S, Tanaka Y, Ishido S, Inada K, Kirino S, Yamashita K *et al*: **Early experience of atezolizumab plus bevacizumab therapy in Japanese patients with unresectable hepatocellular carcinoma in real-world practice**. *INVEST NEW DRUG* 2022, **40**(2):392-402.
41. Xu MH, Huang C, Li ML, Zhu XD, Tan CJ, Zhou J, Fan J, Sun HC, Shen YH: **Effectiveness and safety of lenvatinib plus anti-programmed death-1 antibodies in patients with hepatocellular carcinoma: A real-world cohort study**. *CANCER MED-US* 2023.
42. Choi WM, Lee D, Shim JH, Kim KM, Lim YS, Lee HC, Yoo C, Park SR, Ryu MH, Ryoo BY *et al*: **Effectiveness and safety of nivolumab in child‐pugh b patients with hepatocellular carcinoma: A real-world cohort study**. *CANCERS* 2020, **12**(7):1-14.
43. Xiang YJ, Wang K, Zheng YT, Feng S, Yu HM, Li XW, Cheng X, Cheng YQ, Feng JK, Zhou LP *et al*: **Effects of Stereotactic Body Radiation Therapy Plus PD-1 Inhibitors for Patients With Transarterial Chemoembolization Refractory**. *FRONT ONCOL* 2022, **12**.
44. Sho T, Suda G, Yamamoto Y, Furuya K, Baba M, Ogawa K, Kubo A, Tokuchi Y, Fu Q, Yang Z *et al*: **Efficacy and Effect on Liver Functional Reserve of Atezolizumab and Bevacizumab for Unresectable Hepatocellular Carcinoma in Patients Who Do Not Meet Eligibility Criteria of IMbrave150**. *CANCERS* 2022, **14**(16).
45. Hatanaka T, Kakizaki S, Hiraoka A, Tada T, Hirooka M, Kariyama K, Tani J, Atsukawa M, Takaguchi K, Itobayashi E *et al*: **Prognostic impact of C-reactive protein and alpha-fetoprotein in immunotherapy score in hepatocellular carcinoma patients treated with atezolizumab plus bevacizumab: a multicenter retrospective study**. *HEPATOL INT* 2022, **16**(5):1150-1160.
46. Dong D, Zhu X, Wang H, Li L, Wan M, Li S, Zhang Y, Geng J, Li Y, Wang W: **Prognostic significance of albumin-bilirubin score in patients with unresectable hepatocellular carcinoma undergoing combined immunotherapy and radiotherapy**. *J Med Imaging Radiat Oncol* 2022, **66**(5):662-670.
47. Xiang Y, Wang K, Yu H, Li X, Cheng Y, Wang W, Feng J, Bo M, Qin Y, Zheng Y *et al*: **Transarterial chemoembolization plus a PD-1 inhibitor with or without lenvatinib for intermediate-stage hepatocellular carcinoma**. *HEPATOL RES* 2022, **52**(8):721-729.
